# Enabling high energy lithium metal batteries via single-crystal Ni-rich cathode material co-doping strategy

**DOI:** 10.1038/s41467-022-30020-4

**Published:** 2022-04-28

**Authors:** Xing Ou, Tongchao Liu, Wentao Zhong, Xinming Fan, Xueyi Guo, Xiaojing Huang, Liang Cao, Junhua Hu, Bao Zhang, Yong S. Chu, Guorong Hu, Zhang Lin, Mouad Dahbi, Jones Alami, Khalil Amine, Chenghao Yang, Jun Lu

**Affiliations:** 1 Guangzhou Key Laboratory for Surface Chemistry of Energy Materials, New Energy Research Institute, School of Environment and Energy, South China University of Technology, Guangzhou, 510006 China; 2 School of Metallurgy and Environment, Central South University, Changsha, 410083 China; 3 Chemical Sciences and Engineering Division, Argonne National Laboratory, Lemont, IL 60439 United States; 4 National Synchrotron Light source II, Brookhaven National Laboratory, Upton, NY 11973 United States; 5 School of Materials Science and Engineering, Zhengzhou University, Zhengzhou, 450001 China; 6 Materials Science and Nano-Engineering Department, Mohammed VI Polytechnic University, Ben Guerir, Morocco

**Keywords:** Batteries, Batteries, Transmission electron microscopy, Inorganic chemistry

## Abstract

High-capacity Ni-rich layered oxides are promising cathode materials for secondary lithium-based battery systems. However, their structural instability detrimentally affects the battery performance during cell cycling. Here, we report an Al/Zr co-doped single-crystalline LiNi_0.88_Co_0.09_Mn_0.03_O_2_ (SNCM) cathode material to circumvent the instability issue. We found that soluble Al ions are adequately incorporated in the SNCM lattice while the less soluble Zr ions are prone to aggregate in the outer SNCM surface layer. The synergistic effect of Al/Zr co-doping in SNCM lattice improve the Li-ion mobility, relief the internal strain, and suppress the Li/Ni cation mixing upon cycling at high cut-off voltage. These features improve the cathode rate capability and structural stabilization during prolonged cell cycling. In particular, the Zr-rich surface enables the formation of stable cathode-electrolyte interphase, which prevent SNCM from unwanted reactions with the non-aqueous fluorinated liquid electrolyte solution and avoid Ni dissolution. To prove the practical application of the Al/Zr co-doped SNCM, we assembled a 10.8 Ah pouch cell (using a 100 μm thick Li metal anode) capable of delivering initial specific energy of 504.5 Wh kg^−1^ at 0.1 C and 25 °C.

## Abstract (web summary)

Li-ion cathode active materials are transitioning from poly- to single-crystal structures. However, the performance of high Ni-content single-crystal cathodes remains below expectations. Here, via Al/Zr co-doping, the authors propose a strategy to mitigate structural degradation in this class of materials.

## Introduction

The pressing demand for high specific energy (> 500 Wh kg^−1^) poses challenging requiements on accessible capacity and long cycle life cathode materials used in lithium ion batteries^1–3^. Among the current state-of-the-art commerical cathodes, LiNi_*x*_Co_*y*_Mn_1−*x−y*_O_2_ (NCM) layered oxide cathode, in particular, Ni-rich NCM (Ni content ≥ 60 mol%) is leading a technologically significant development trend of cathode materials by virtue of its high capacity, low cost and improved kinetic performance^4,5^. Despite these merits, Ni-rich NCM usually suffers from structural instability and capacity degradation during cycling, which restricts its large-scale commercial application^6^. To overcome or circumvent these issues, scientific researchers devoted substantial efforts to understand the origin of the fast capacity decay of Ni-rich NCM. A clear consensus has been reached that its fast capacity decay is predominantly rooted in surface chemical reactions and particle cracking^7^. With Ni content increasing, NCM undergoes a large anisotropic volume change, particularly the rapid lattice collapse at high delithiation states, which affects the structural integrity and causes chemo-mechanical degradation in the form of intergranular cracks^8^. These intergranular cracks will propagate along the grain boundaries during cycling, aggravating the pulverization and capacity degradation of Ni-rich NCM^9^. In addition, the non-aqueous organic electrolyte liquid solution used for cell cycling could penetrate into Ni-rich NCM microspheres along the intergranular cracks, resulting in the global occurrence of surface parasitic reaction and structural degradation as well as the capacity fading^10–13^.

One promising approach to mitigate these issues is to develop single-crystal NCM, which recently has attracted increasing attention both in industry and academia^14,15^. Benefiting from the enhanced structural integrity and boundary-free configuration, single-crystal LiNi_*x*_Co_*y*_Mn_1−*x−y*_O_2_ (*x* ≤ 0.7) has been demonstrated to effectively suppress particle cracking and limit electrolyte penetration, leading to a significant improvement in capacity retention and comprehensive advantages aginst conventional polycrystalline battery particles^16–18^. However, the advancement in cycle stability of these single-crystal cathodes comes at the expense of reversible capacity and rate capability, owing to the limited Ni content and low Li^+^ diffusion efficiency in the micron-scale particles ^18,19^.

To break these confinements, extending the success of existing single crystals to higher Ni content with high voltage functionality has been considered as a straightforward strategy, but it has not yet been much reported becasue of several challenges faced by Ni-rich single crystal cathode. On the one hand, high Ni component will increase the difficulty of synthesizing stoichiometric single crystals with competitive electrochemical energy storage properties. Likewise, Ni-rich single crystal is susceptible to the increased Li/Ni disorder. This negative affects Li^+^ diffusion efficiency and reversible capacity^20,21^. On the other hand, increasing Ni content will substantially aggravate the phase transition from the second hexagonal phase (H2) to the third hexagonal phase (H3) accompanied by an abrupt lattice contraction of Ni-rich NCM at high delithiation states^22,23^. This will continuously build up internal strains during extended cycling, eventually resulting in the generation of nanocracks (the size of nanocrack is generally less than 20 nm)^24^. In addition, most of Ni ions in the Ni-rich single crystal will be oxidized to Ni^4+^ ions at high delithiation states, but Ni^4+^ ions are thermodynamically metastable and tend to be reduced to more stable Ni^2+^ ions with the formation of NiO rock-salt on the NCM surface^25,26^. This partial reconstruction with the formation of NiO and Li/Ni^2+^ mixing would hinder the Li^+^ migration, which can extend from the surface to interior bulk, eventually resulting in the performance degradation of NCM electrode. It will trigger the harmful phase transformation from layered to spinel/rock-salt phase^26,27^. With all things considering, creative strategies to solve the above deficiencies are necessary to achieve satisfactory electrochemical properties and accelerate the large commercial application of Ni-rich NCM single-crystals.

In this work, we report that trace amounts of Al/Zr concurrent-doping (0.3 mol% of Al or Zr) can enhance the dynamic structure reversibility and Li^+^ diffusion mobility of single-crystalline LiNi_0.88_Co_0.09_Mn_0.03_O_2_ (SNCM) cathode materials. Generally, the solubility difference among various doping elements will easily induce the different deposition behaviors during the in situ doping co-precipitation, resulting in that partial doping element are incompletely substituted into the SNCM lattice during the sintering process with the formation of residue coating layer^27^. Especially for Zr-doping, it is inclined to generate a layer Li_2_ZrO_3_ on the NCM surface. Therefore, according to element precise control and experimental condition optimization, the doped Al ions are well incorporated its parent lattice of SNCM single crystal, while the high-valence Zr ions with limited solubility incline to aggregate in the outer surface layer of SNCM single crystals by adjusting the precursor feeding sequence. Combining comprehensive electrochemical measurements and multiscale structural/morphological characterizations, we reveal that the synergistic effect of Al/Zr co-doping in SNCM lattice can effectively enhance the Li^+^ diffusion mobility, suppress Li/Ni cation mixing, and release the internal strain upon cycling at 4.6 V, which contributes to much improved structural stability and electrochemical performance. Meanwhile, the Zr-rich surface benefits to protect the SNCM from parasitic reactions induced by the organic electrolyte and inhibit irreversible phase transition. As a result, the designed Al/Zr co-doped SNCM could simultaneously deliver a specific capacity over 221 mAh g^−1^, good rate capability up to 5 C (i.e., 1 A g^−1^ considering the mass of the positive electrode active material for the coin-type half-cell measurements) and improved cycle performance at coin-cell level. More importantly, to demonstrate the practicality of this modification, we fabricate 10.83 Ah Ni-rich single crystal||Li metal pouch cells that exhibit a specific energy of 504 Wh kg^−1^ (calculated considering the mass of the whole pouch cell components) at 0.1 C (i.e., 20 mA g^−1^ considering the mass of the positive electrode active material for the pouch-type full-cell measurements) and 25 °C.

## Results

### Fabrication and physicochemical characterizations of pristine and doped SNCM

**Fig. 1:**
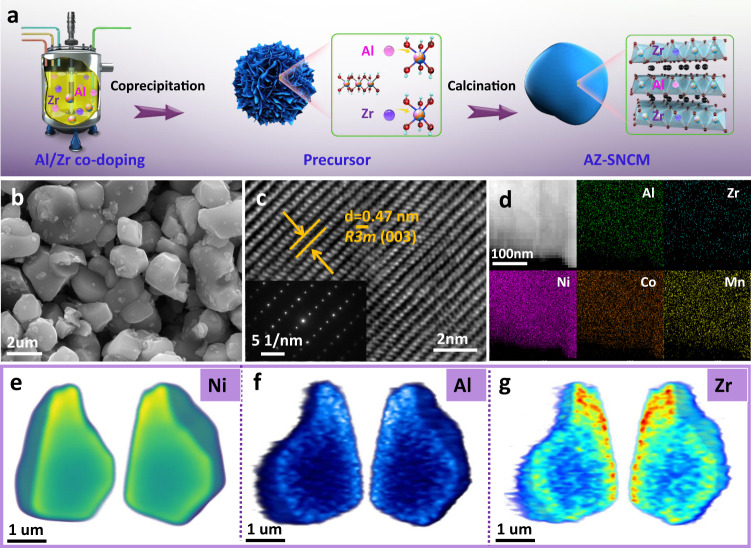
Synthetic process, morphology, and chemical composition of the AZ0.3-SNCM. **a** Schematic illustration of the synthesis process of AZ0.3-SNCM. SEM (**b**), HRTEM (**c**) images and SAED pattern (inset) for AZ0.3-SNCM. EDS elemental mappings of Ni, Co, Mn, Al, Zr, and O elements (**d**). Mesoscale element heterogeneity revealed by nano-resolution 3D X-ray fluorescence. The 3D spatial distribution of Ni (**e**), Al (**f**), and Zr (**g**) in the AZ0.3-SNCM single crystal.

LiNi_0.874_Co_0.09_Mn_0.03_Al_0.003_Zr_0.003_O_2_ (AZ0.3-SNCM) single crystals are prepared by the combination of co-precipitation and calcination process. As shown in Fig. 1a, raw materials and doping sources are pumped into the reaction tank with continuous purging with N_2_ gas to remove the O_2_^28^. Trace amounts of Al/Zr will be added during the co-precipitation process and form Ni_0.874_Co_0.09_Mn_0.03_Al_0.003_Zr_0.003_(OH)_2_ precursors, which are consisted of porous spherical secondary flowers with a diameter of 4–5 μm (Supplementary Fig. 1). Interestingly, after the modified solid-state calcination reaction at high temperature (820 °C) and grinding into micron-sized particles, the precursors are converted into quasi single-crystal primary particles (Supplementary Fig. 2). The actual chemical composition of as-prepared AZ0.3-SNCM is directly inherited from the in situ co-doped precursors, which is confirmed by the inductively coupled plasma (ICP) elemental analysis (Supplementary Table 1). To better understand the roles of Al and Zr on the structural/electrochemical properties, undoped SCNM, 0.3 mol% Al-doped SNCM (Al-SNCM), 0.3 mol% Zr doped SNCM (Zr-SNCM), 0.6 mol% or 3.0 mol% Al/Zr co-doped SNCM (AZ0.6-SNCM or AZ3.0-SNCM) have been prepared by the similar method, respectively.

AZ0.3-SNCM demonstrates a well-defined single-crystalline morphology with a particle size of 2–5 μm by scanning electron microscopy (SEM, Fig. 1b). Rietveld refinements of XRD patterns indicate that AZ0.3-SNCM exhibits a pure hexagonal α-NaFeO_2_ structure with the R$\bar{3}$m space group (Supplementary Fig. 3). Compared with undoped SNCM, the *c* lattice parameter of AZ0.3-SNCM increases from 14.207 Å (Supplementary Table 2) to 14.210 Å (Supplementary Table 3), in accordance with the slight shift of (003) peaks to lower angle (Supplementary Fig. 4), which is attributed to the larger ionic radius of doped Zr ions than that of Ni ions^29^. Morphology and fine structure of AZ0.3-SNCM have been further investigated via transmission electron microscopy (TEM). Supplementary Fig. 5a–d displays a typical quasi single-crystalline primary particle of AZ0.3-SNCM with a diameter of 4 μm, which is different from the conventional Ni-rich NCM secondary particles^25^. High-resolution TEM (HRTEM) image of AZ0.3-SNCM is displayed in Fig. 1c, and the lattice space of 0.47 nm is corresponded to the (003) plane of layered structure SNCM, which is verified by the selected area electron diffraction (SAED) pattern (inset of Fig. 1c). Furthermore, the AZ0.3-SNCM sample exhibit smooth edges without obvious coating layer, confirming the successful substitution of Al/Zr into the bulk structure (Supplementary Fig. 5e, f). Energy-dispersive X-ray spectroscopy (EDS) elemental mapping results (Fig. 1d) indicate that the Al and Zr ions have been successfully doped in single crystal particles. In consideration of the limited resolution of elemental mapping, three-dimensional X-ray fluorescence tomography (3D XRF) was conducted to quantitatively analyze the distribution of Al and Zr foreign dopants. Similar with the 3D renderings of homogeneous Ni element (Fig. 1e), Al is evenly distributed throughout the entire AZ0.3-SNCM single crystal (Fig. 1f), indicating it high solubility in SNCM. By contrast, Zr is preferably concentrated in the outer surface layer of AZ0.3-SNCM single crystal with obvious aggregation (Fig. 1g). The gradually decreased intensity of Zr from the outer surface layer to the interior of AZ0.3-SNCM single crystal companied with the relative homogeneous distribution of Al are further confirmed by in-depth EDS spectra (Supplementary Fig. 6) and X-ray photoelectron spectroscopy (XPS, Supplementary Fig. 7), which is consistent with what was initially designed.

### Electrochemical energy storage properties of the doped SNCM materials

**Fig. 2:**
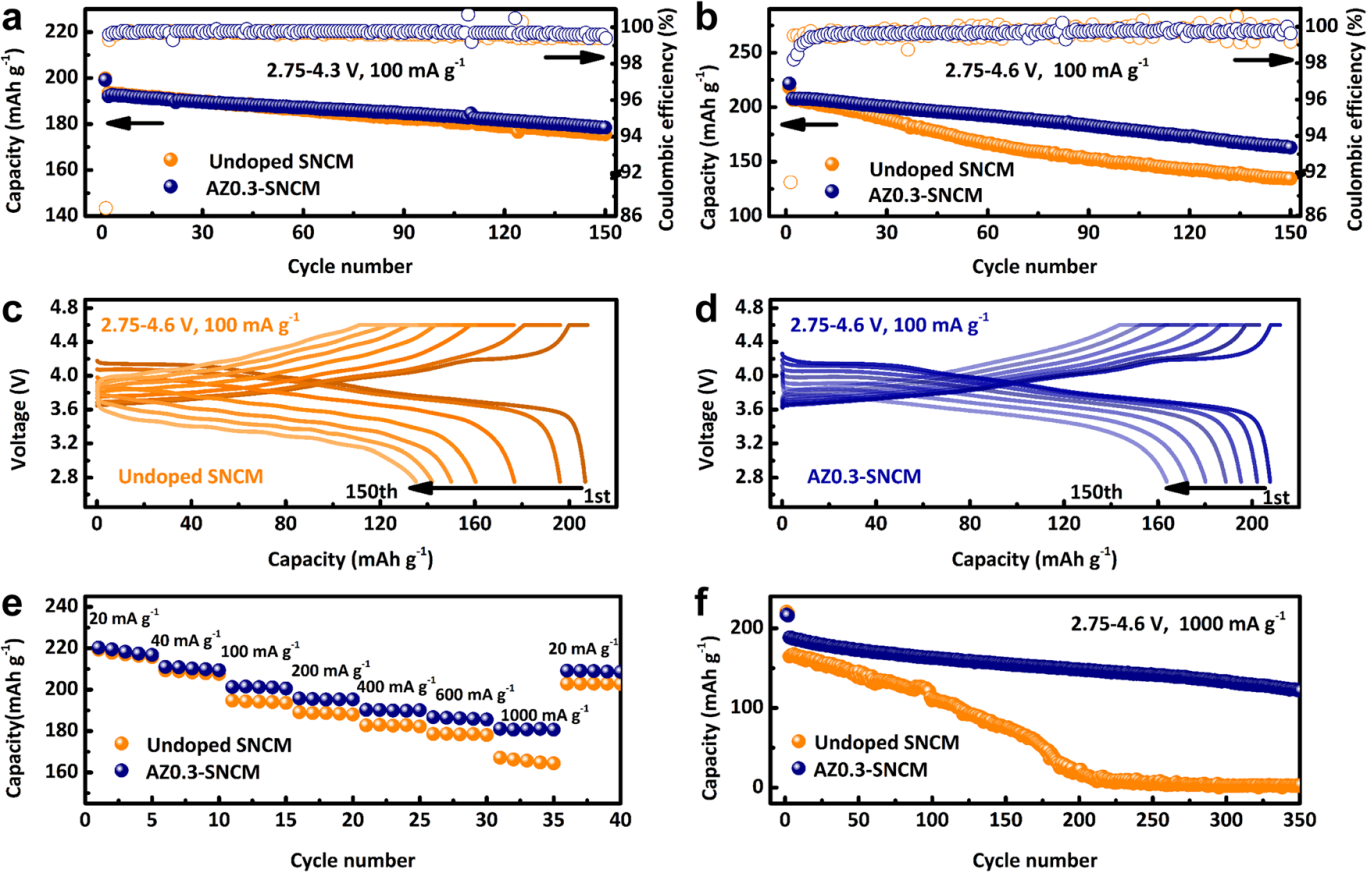
Electrochemical characterizations of coin-type half-cell at 25 °C. (Li metal anode and 1 M LiPF_6_ in EC/DEC (1:1 in volume) are used as the counter electrode and electrolyte, respectively.) Cycling stability for undoped SNCM and AZ0.3-SNCM within the voltage range of 2.75–4.3 V (**a**) and 2.75–4.6 V (**b**) at 0.5 C (100 mA g^−1^). Charge/discharge curves for undoped SNCM (**c**) and AZ0.3-SNCM (**d**) at 0.5 C (100 mA g^−1^) within the voltage range of 2.75–4.6 V. Rate performance (**e**) and cycling stability (**f**) for SNCM and AZ0.3-SNCM at 5 C (1000 mA g^−1^) within the voltage range of 2.75–4.6 V, respectively.

To understand the influence of trace amounts of Al/Zr dual-doping on the ability of SNCM to efficiently store Li ions, the electrochemical properties of SNCM and doped SNCM samples have been investigated by coin-type cells with as-prepared cathodes and Li metal anode in the electrolyte of 1 M LiPF_6_ in ethyl carbonate/diethylene carbonate (EC/DEC, 1:1 in volume) initially. AZ0.3-SNCM demonstrates the best cycling performance, especially at the high cut-off voltage (Supplementary Fig. 8). As presented in Fig. 2a, both undoped SNCM and AZ0.3-SNCM exhibit a similar initial discharge capacity of 193 mAh g^−1^ at 0.5 C (100 mA g^−1^) within the voltage range of 2.75–4.3 V (note that all cells were cycled at 20 mA g^−1^ in the first cycle for activation). After 150 cycles at 0.5 C (100 mA g^−1^), the reversible capacity of AZ0.3-SNCM is slightly higher than undoped SNCM. Whereas, the difference of reversible capacity between undoped SNCM and AZ0.3-SNCM maintains small during 250 cycles (Supplementary Fig. 9). Notably, compared with AZ0.3-SNCM, undoped SNCM exhibits a distinct capacity degradation after elevating the cut-off voltage to 4.4 V. As exhibited in Supplementary Fig. 10a, AZ0.3-SNCM exhibits a reversible discharge capacity of 162.3 mAh g^−1^ at 0.5 C (100 mA g^−1^) after 150 cycles with a capacity retention of 83.4%, which is higher than that of undoped SNCM for 140.9 mAh g^−1^. When the cut-off voltage increased to 4.6 V, AZ0.3-SNCM displays a high discharge capacity of 221.6 mAh g^−1^ at 0.1 C (20 mA g^−1^), and it can still achieve a reversible capacity of 163.0 mAh g^−1^ at 0.5 C (100 mA g^−1^) after 150 cycles (Fig. 2b). However, the discharge capacity of undoped SNCM degrades fast to 134.4 mAh g^−1^ at 0.5 C (100 mA g^−1^) after 150 cycles. Selected charge/discharge profiles of undoped SNCM and AZ0.3-SNCM are presented in Fig. 2c, d. The average voltage of AZ0.3-SNCM retains above 3.58 V at 0.5 C (100 mA g^−1^) after 150 cycles within 2.75–4.6 V, while that of undoped SNCM drops to only 3.33 V.

Figure 2e compares the rate capability of undoped SNCM and AZ0.3-SNCM. It is evident that AZ0.3-SNCM consistently outperforms the undoped SNCM counterpart and still delivers a reversible discharge capacity of 181.1 mAh g^−1^ after a 50-folds increase in the current rate from 0.1 to 5.0 C within a cut-off voltage of 4.6 V (82.2% of the capacity at rate of 100 mA g^−1^). When the current rate is reset back to 0.1 C (20 mA g^−1^), the capacity of AZ0.3-SNCM is almost fully recovered. Besides, AZ0.3-SNCM can still deliver a discharge capacity of 121.2 mAh g^−1^ at 5.0 C (1000 mA g^−1^) with a cut-off voltage of 4.6 V after 350 cycles, while, that of undoped SNCM drops to merely 2.8 mAh g^−1^ (Fig. 2f and Supplementary Fig. 10b). Unprecedentedly, even at the high-temperature of 55 °C and cut-off voltage of 4.6 V, AZ0.3-SNCM still exhibits a good cycling stability (Supplementary Fig. 11). AZ0.3-SNCM can deliver an initial discharge capacity of 234.8 mAh g^−1^ at 0.1 C (20 mA g^−1^) with coulombic efficiency of 88.2%, and a discharge capacity of 183.6 mAh g^−1^ at 0.5 C (100 mA g^−1^) after 50 cycles. The improved thermal stability is also confirmed by the higher exothermic peak of delithiated AZ0.3-SNCM cathode (Supplementary Fig. 12). Compared to undoped SNCM, AZ0.3-SNCM exhibits a suppressed voltage fade and improved cycling performance, supporting the speculation about the improved structural stability of the doped materials.

**Fig. 3:**
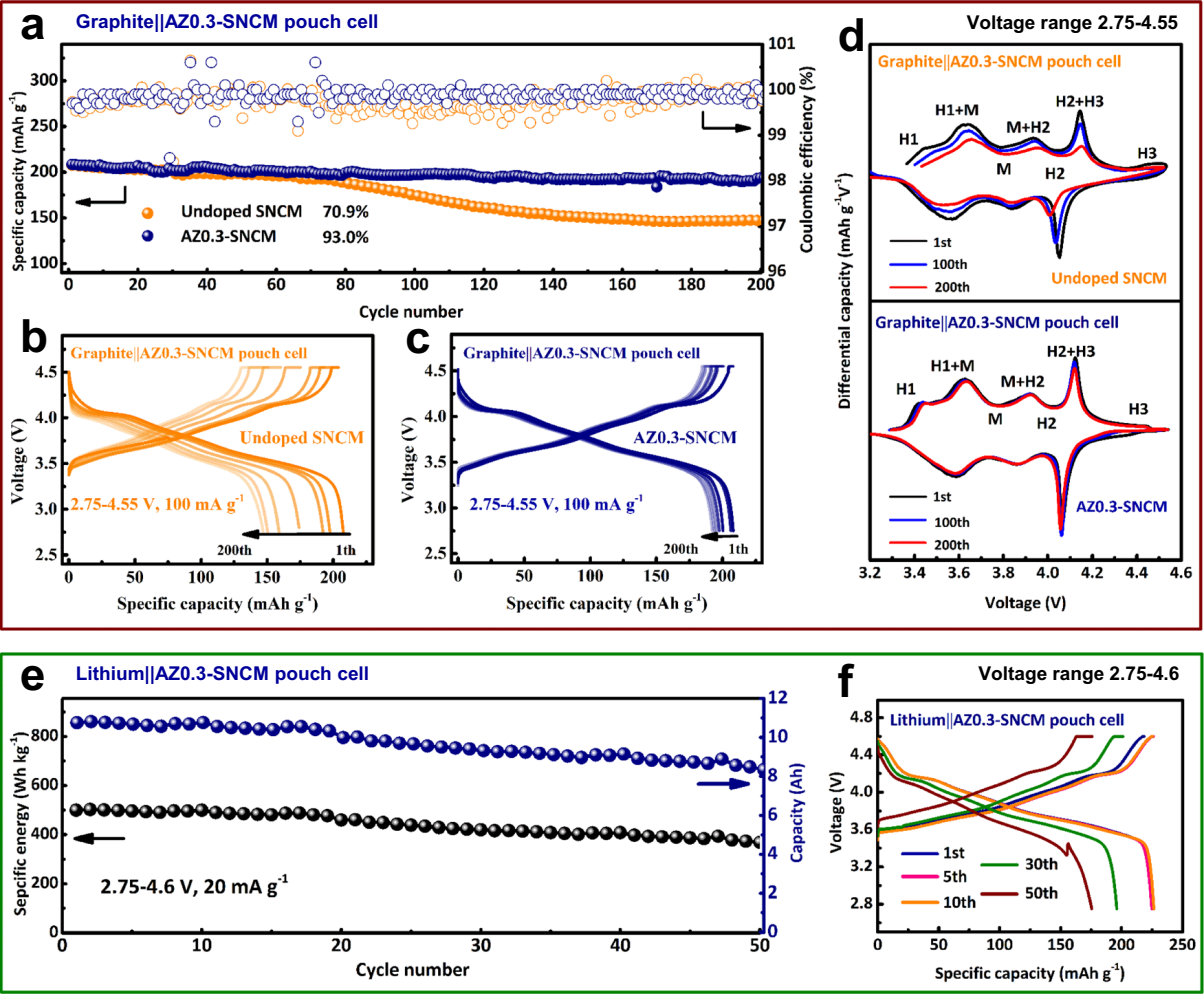
Electrochemical characterization of pouch-type full-cell at 25 °C. (1.1 M LiPF_6_ in ethyl carbonate/ethyl methyl carbonate/diethyl carbonate (EC/EMC/DEC, 3:5:2 in volume) + 1 wt% vinylene carbonate is used as the electrolyte)**. a** Cycling performance of full cells with undoped SNCM or AZ0.3-SNCM as cathode and graphite as anode at 0.5 C (100 mA g^−1^) within the voltage range of 2.75–4.55 V. Charge/discharge curves for undoped SNCM (**b**) and AZ0.3-SNCM (**c**) at different cycles. **d** The d*Q* d*V*^−1^ curves for undoped SNCM and AZ0.3-SNCM at various cycles. Cycling property (**e**) and corresponding charge/discharge curves (**f**) of AZ0.3-SNCM||Li foil pouch full cells within the potential window of 2.75–4.6 V.

For practical applications evaluation, pouch-type full cells (made by double-coated electrodes and assembled by 8 electrodes layers) with as-prepared cathodes and commercial graphite anode were assembled and cycled within the voltage range of 2.75–4.55 V (equivalent to 4.6 V versus Li/Li^+^), as shown in Fig. 3a. It is noted the practical mass cathode loading of 31 mg cm^−2^ is used for these pouch-type full cell tests, which is significantly higher than the laboratory level and comparable to the commercial production^29,30^. The discharge capacity of SNCM quickly drops to 147.1 mAh g^−1^ at 0.5 C (100 mA g^−1^) after 200 cycles accompanied with a detrimental voltage fade (Fig. 3b). In contrast, the AZ0.3-SNCM based pouch-type full-cell delivers an initial discharge capacity of 208.4 mAh g^−1^ at 0.5 C (100 mA g^−1^), and it can maintain a discharge capacity of 193.8 mAh g^−1^ after 200 cycles with a capacity retention of 93% (Fig. 3c). The corresponding differential capacity (d*Q* d*V*^−1^) profiles during the long-term cycling are illustrated in Fig. 3d. It is clear that undoped SNCM undergoes a series of phase transitions from hexagonal 1 to monoclinic (H1 → M), to hexagonal 2 (M → H2), to hexagonal 3 (H2 → H3) during the lithiation process, and follows a reverse sequence during delithation^24,30^. As the H2 → H3 phase transition will result in an abrupt contraction of the unit cell along the *c*-axis, the cathode materials suffer from a severe mechanical strain with anisotropic volume change^31^. It is noted that the intensity of the peak corresponding to detrimental H2 → H3 phase transition in AZ0.3-SNCM becomes weaker than that of undoped SNCM, illustrating that effectively suppressed H2 → H3 phase transition in AZ0.3-SNCM^32^. In the subsequently cycles, the cathodic peaks of undoped SNCM, especially for the H3 → H2 reduction peak, gradually shift to the lower voltage range accompanied with decreased intensity. By contrast, distinctive cathodic peaks can still be observed for AZ0.3-SNCM after 200 cycles without any significant degradations, indicating a low polarization and improved H2 → H3 phase transition reversibility of AZ0.3-SNCM. It is assigned to the synergistic effect of trace Al/Zr co-doping in bulk SNCM, which can effectively suppress the Li/Ni cation mixing and detrimental phase transition.

Notably, high energy Li-metal pouch cells coupled with a high areal capacity of AZ0.3-SNCM (12.6 mAh cm^−2^) have been fabricated to evaluate the future applications of SNCM||Li-metal battery systems (made by double-coated electrodes and assembled by 12 electrodes layers). The AZ0.3-SNCM||Li-metal pouch cell (2.04 Ah) exhibits a specific energy of 410 Wh kg^−1^ with a capacity retention of 90% after 100 cycles in the voltage range of 2.75–4.6 V (Supplementary Fig. 13). Surprisingly, when the pouch cell is designed with increased mass loading of cathode for higher pack capacity (10.83 Ah), it can display a specific capacity of 225 mAh g^−1^ at 0.1 C (20 mA g^−1^) within 2.75–4.6 V (Fig. 3e and Supplementary Fig. 14), achieving a specific energy of 504.5 Wh kg^−1^ for the first cycle based on the whole pouch cell (including AZ0.3-SNCM cathode materials, Li-metal anode, electrolyte, separators, aluminum foil and aluminum jacket et al.). Its specific energy retention is about 73.7% after 50 cycles, which can be improved by the pouch-type full-cell configuration design and electrolyte optimization. In comparison with the practical LIBs pouch full-cell system (with commercial LiCoO_2_ (LCO), NCM, or NCA et al. as cathodes) as reported elsewhere, the rational designed AZ0.3-SNCM (whether using the graphite or Li-metal as anode) in this work demonstrates an improved battery performance (Supplementary Table 4) ^18,30,31,33–36^.

### The role of Al and Zr on the SNCM electrochemical energy storage behavior and structural stability

**Fig. 4:**
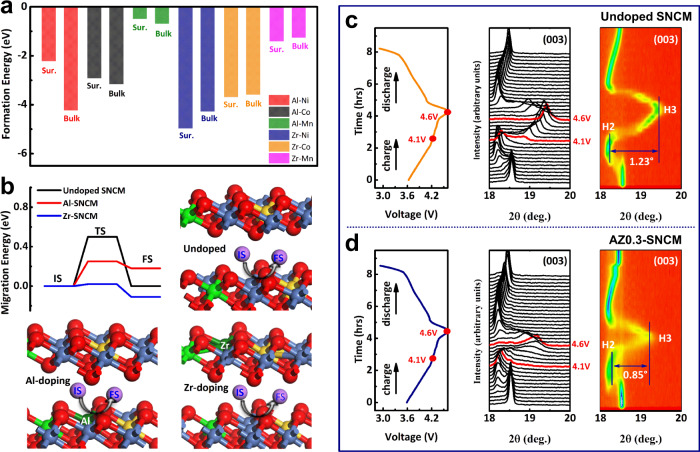
Density theory calculation and in situ XRD characterization during charge/discharge process. **a** Formation energy for replacing Ni, Co, or Mn ions both surface (Sur.) and bulk layer (Bulk) with Al or Zr ions. **b** Migration energy and corresponding schematic structure for undoped SNCM, Al or Zr doped SNCM (Al-SNCM and Zr-SNCM): IS, TS and FS represent initial state, transition state, and final state, respectively. In situ XRD characterizations of undoped SNCM (**c**) and AZ0.3-SNCM (**d**) during the initial charge–discharge cycle. In situ XRD measurement was conducted on an in-house developed cell with Li metal and 1 M LiPF_6_ in EC/DEC (1:1 in volume) as counter electrode and electrolyte at 25 °C.

To better understand the enhanced structural integrity of AZ0.3-SNCM, density functional theory (DFT) calculations have been conducted to estimate the formation energies between Al/Zr and Ni, Co, or Mn ions in SNCM. First, Al and Zr ions are considered to replace Ni, Co, or Mn ions both on the surface and in bulk SNCM, respectively, and the corresponding formation energies are listed in Fig. 4a. It is noticed that Al and Zr ions prefer to replace Ni ions in SNCM. The Al ions with high solubility in SNCM are well incorporated its host lattice, exhibiting a lower formation energy of −4.23 eV. But the Zr ions in bulk SNCM are structurally unstable and part of them incline to aggregate on the outer surface layer of SNCM single crystals (Supplementary Fig. 15), it indicates that the conductivity of both Al-SNCM and Zr-SNCM are much higher than that of pristine SNCM. Moreover, Al/Zr co-doping effect on the migration energy barrier of Li^+^ diffusion in bulk SNCM have also been calculated. As shown in Fig. 4b, the Li^+^ migrate energy barrier for undoped SNCM is 500 meV, and it reduces to 70 and 130 meV by Al-doing and Zr-doping, respectively. For Li migration pathway (Supplementary Fig. 16), it is seen that the Li ion will move to the bridge site between the two O atoms and trend to migrate to another nearest hollow site (final state). For the migration of Ni (Supplementary Fig. 17), Ni atom in the bulk phase will be out of the internal layer (initial state) and move to gap between the two layers (finial state). It is seen that the barrier for Ni atom migration in undoped SNCM is 2.07 eV, which is lower than those of Zr-doped (2.37 eV) and Al-doped (2.68 eV) configuration. This suggests that the Ni will be more stable after Zr or Al atom being doped. The Li^+^ diffusion coefficient in AZ0.3-SNCM has been experimentally determined via electrochemical impedance spectroscopy (EIS) measurement (Supplementary Fig. 18a). Supplementary Table 5 shows that the Li^+^ diffusion coefficient in AZ0.3-SNCM is much higher than that of undoped SNCM, confirming the synergistic effect of trace Al/Zr co-doping on the enhanced Li^+^ ions diffusion mobility as well as improved rate capability ^37,38^.

Structural evolution of AZ0.3-SNCM during lithiation/delithiation process has been investigated. In situ XRD measurement was conducted on an in-house developed cell with undoped SNCM or AZ0.3-SNCM as cathode cycled at 0.1 C (20 mA g^−1^) within 2.75–4.6 V, and the collected in situ XRD patterns and two-dimensional (2D) contour plots are compared. As presented in Fig. 4c, when undoped SNCM cathode is charged from 2.75 to 4.1 V, the (003) diffraction peak of SNCM shifts to the lower 2θ, indicating that *c*-axis gradually expands along with a phase transformation from H1 to M caused by electrostatic repulsion between adjacent oxygen layers. Afterwards, the (003) diffraction peak splits into two peaks, which are associated with the H2–H3 phase transition. When it is fully charged to 4.6 V, the (003) diffraction peak undergoes an aggressive shift to the higher 2θ, demonstrating a drastically lattice contraction along the *c*-axis during the H2–H3 phase transition^24,26^. It is noticed that the (003) diffraction peak of undoped SNCM contains a broad shoulder, which is mainly assigned to H2 and H3 two-phase separation transition induced by the Li^+^/vacancy ordering within Li-layers, resulting in the severe irreversible H2–H3 phase transition^26,39,40^. While, the phase transformation process of AZ0.3-SNCM is alike but different to that of undoped SNCM. As indicated in Fig. 4d, the H2–H3 transition can still be observed, the almost identical shape of (003) diffraction peak demonstrates a pseudo single-phase H2–H3 transition at the high cut-off voltage of 4.6 V with reversible structural evolution. This result reveals that the Al/Zr co-doping in bulk SNCM can effectively alleviate the electronic redistribution with lower Li^+^/vacancy ordering, suppress the undesired two-phase separation, and enhance the H2–H3 phase transition reversibility.

The shift of (003) diffraction peak to higher 2θ for SNCM cathode materials accompanied with the decrease of the *c* parameter indicate the decreased interslab distance at the highly delithiated state. This phenomenon is ascribed to that the decreased electrostatic repulsion between the transition-metal layers can lead to the enhanced covalency of Ni^4+^–O^2−^ bonds. But the continuous removal of Li^+^ ions at the highly delithiated state between the transition-metal layers can easily destroy the pillaring effect and induce structure collapse with abrupt variation in the interslab distance. As shown in Fig. 4c, d, there is an apparent distinction between the (003) diffraction peak for SNCM and AZ0.3-SNCM at the end of charging process. The (003) diffraction peak of AZ0.3-SNCM shifts right by 0.85° during H2–H3 transition (Fig. 4d), which is smaller than that of undoped SNCM that shifts right by 1.13° (Fig. 4c). It indicates that the shrinkage rate of AZ0.3-SNCM along the *c*-axis is reduced 0.7% compared to undoped SNCM (Supplementary Fig. 19). The remarkable improvement in reduced lattice contraction is assigned to the pillaring effect of doped Al/Zr ions in SNCM, which can effectively release the internal-strain and impede the local structure collapse. Furthermore, the doped Al/Zr ions can further enhanced covalency of Ni^4+^–O^2−^ bonds (Supplementary Fig. 20), prevent the reduction of highly oxidized Ni^4+^ to Ni^2+^ and migration of Ni^2+^ into Li-layer, thus inhibiting the anisotropic volume variation and strengthening the crystal stability^41,42^. Based on the comparison, we can verify that the synergistic effect of trace Al/Zr co-doping in SNCM can serve as pillars to effectively release the internal-strain and enhance the structural stability at 4.6 V.

### Cationic redox and side reaction in SNCM cathodes

**Fig. 5:**
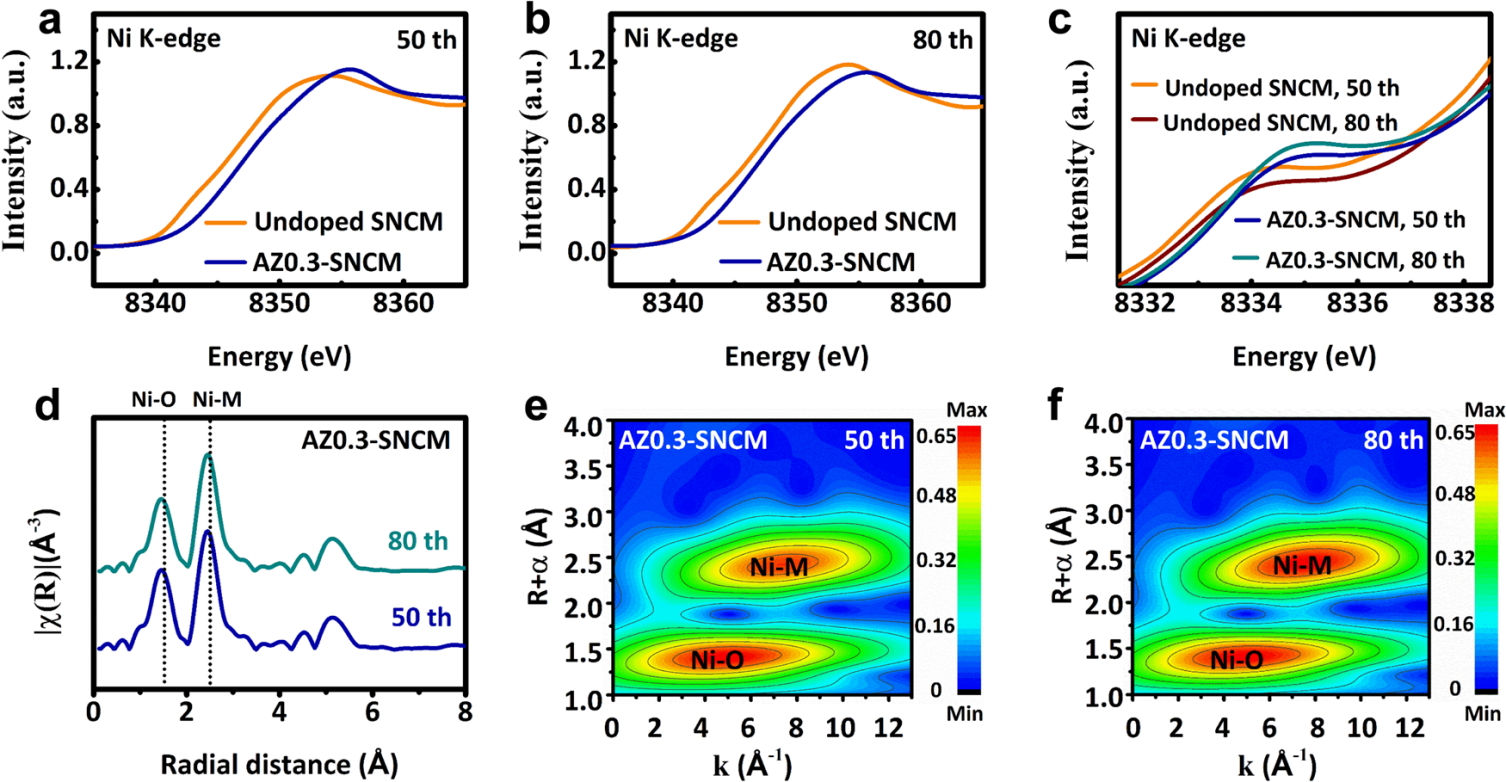
Ex situ chemical characterizations of the SNCM electrodes after 80 cycles at 0.5 C (100 mA g^−1^) and a cut-off voltage of 4.6 V. **a**, **b** XANES spectra of Ni K-edge of undoped SNCM and AZ0.3-SNCM cathodes collected at fully charged state after various cycles. **c** XANES derivative plots for pre-edge peaks for undoped SNCM and AZ0.3-SNCMcathodes. **d** Ex situ Ni K-edge EXAFS of AZ0.3-SNCM cathode after various cycles. **e**, **f** 2D Fourier transformed Ni K-edge EXAFS spectra for AZ0.3-SNCM cathodes after 50 and 80 cycles.

The fact that Al/Zr co-doping prevents lattice contraction at high voltage will affect the Ni redox in SNCM single crystals. Although higher Ni content can increase the specific capacity of cathode materials, this is unfortunately accompanied by the irreversible reduction of Ni ions into Ni^2+^ ions at highly delithiated state. These Ni^2+^ ions further migrate into Li layers, leading to Li/Ni cation mixing and phase transition from layer structure to rock-salt phase. Therefore, X-ray absorption near edge structure (XANES) spectroscopy has been employed to study the Ni chemical valence state in undoped SNCM and AZ0.3-SNCM during cycling^23,43,44^. As indicated in Fig. 5a, b, the Ni K-edges for undoped SNCM under fully charged state keep energy position of 8354.1 eV from 50th to 80 cycles without phase correction, indicating the relatively decreased oxidation state for Ni ions due to Ni^3+^/Ni^4+^ redox^45^. While, we can observe that the Ni K-edges for cycled AZ0.3-SNCM exhibit slight left shift, yet its energy (~8355.0 eV) is higher than that of undoped SNCM along with cycling, demonstrating that the cycled AZ0.3-SNCM particles retain high Ni^3+^/Ni^4+^ concentration. It demonstrates that the oxidation state for Ni in AZ0.3-SNCM is much higher than that in undoped SNCM with better utilization and reversibility of Ni^3+^/Ni^4+^ redox, owing to the robust layered structure after Al/Zr doping^46,47^. Furthermore, the white line intensity of cycled AZ0.3-SNCM is lower than that of undoped SNCM, which is presumably ascribed to the accumulation of Ni^2+^ compounds on the surface of undoped SNCM, despite that the Ni^4+^ presents as the main oxidized state in the interior bulk. Such combination results in the average Ni valence of undoped SNCM is lower than that in AZ0.3-SNCM, which is consistent with the pre-edge results (Fig. 5c) ^45^.

The cycling condition at the high charging cut-off voltage can trigger the side reaction between cathode materials and organic electrolyte, resulting in the continuous dissolution of Ni ions from SNCM. Thus, the extended X-ray absorption fine structure (EXAFS) analysis which are more sensitive to local chemical and structure changes has been performed to directly investigate the local environment of Ni upon cycling^48,49^. The EXAFS analysis demonstrates that two local environments of Ni–O and Ni–M are observed for undoped SNCM. It is noticed that both the Ni–O and Ni–M interatomic distances for undoped SNCM are lengthened after 80 cycles (Supplementary Fig. 21) with higher peak intensity, illustrating the excess of Ni^2+^ in the AZ0.3-SNCM owing to the surface deterioration. Interestingly, the Ni–O and Ni–M interatomic distances of Ni keep almost unchanged in AZ0.3-SNCM after 80 cycles (Fig. 5d), suggesting a stable valance state and lattice structure of AZ0.3-SNCM^45,50^. While, the 2D contour Fourier-transformed Ni K-edge EXAFS analysis was carried out to distinguish the backscattering atoms and analyze the Ni local environment of undoped SNCM and AZ0.3-SNCM. Compared with that of undoped SNCM (Supplementary Fig. 22), the Ni–O and Ni–M interatomic distances and intensity for AZ0.3-SNCM remain stable after 50 (Fig. 5e) and 80 cycles (Fig. 5f) even at a high cut-off voltage of 4.6 V, indicating the good stability and reversibility of local coordination environment.

The corresponding Ni *2p* XPS spectra on the surface of undoped SNCM and AZ0.3-SNCM after 150 cycles have been studied and obtained with increasing of Ar^+^ etching from XPS depth profiles of 0, 100, and 300 nm, the results are shown in Supplementary Fig. 23. It is clear that AZ0.3-SNCM exhibits higher concentration of Ni^3+^ compared to undoped SNCM. When combined with XANES and XPS depth profiles, these testing results verify the good reversibility of Ni^3+^/Ni^4+^ redox couple in bulk with limited surface degradation in AZ0.3-SNCM after cycling within 2.75–4.6 V^45,47^, which will eventually mitigate the generation and migration of Ni^2+^ into Li-layer and reduce the Li/Ni cation mixing in AZ0.3-SNCM.

**Fig. 6:**
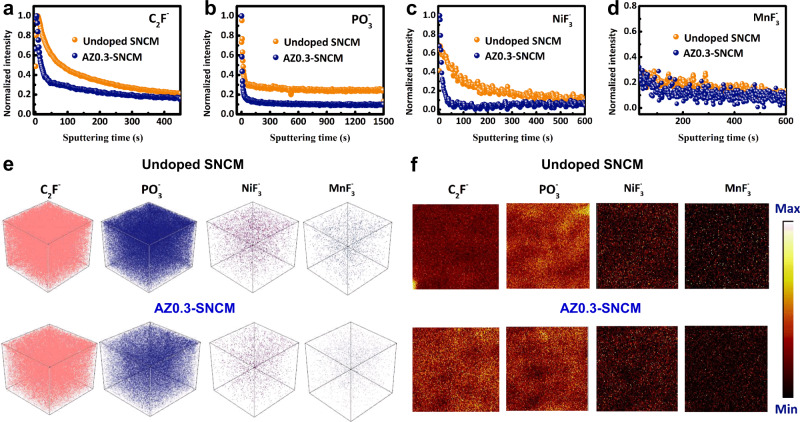
Ex situ chemical characterizations of the SNCM electrodes after 150 cycles at 0.5 C (100 mA g^−1^) within 2.75–4.6 V. TOF-SIMS depth profiles of C_2_F^−^ (**a**), ${{{{{{\rm{PO}}}}}}}_{3}^{-}$ (**b**), ${{{{{{\rm{NiF}}}}}}}_{3}^{-}$ (**c**) and ${{{{{{\rm{MnF}}}}}}}_{3}^{-}$ (**d**) species. 3D render of composition (C_2_F^−^, ${{{{{{\rm{PO}}}}}}}_{3}^{-}$, ${{{{{{\rm{NiF}}}}}}}_{3}^{-}$, and ${{{{{{\rm{MnF}}}}}}}_{3}^{-}$) and concentration distribution (**e**) and TOF-SIMS chemical imaging of C_2_F^−^, ${{{{{{\rm{PO}}}}}}}_{3}^{-}$, ${{{{{{\rm{NiF}}}}}}}_{3}^{-}$, and ${{{{{{\rm{MnF}}}}}}}_{3}^{-}$ species (**f**) on the SNCM and AZ0.3-SNCM cathode surface.

Time-of-flight secondary-ion mass spectrometry (TOF-SIMS) analysis has also been conducted to further elucidate the surface compositions of undoped SNCM and AZ0.3-SNCM after 150 cycles at 0.5 C (100 mA g^−1^) within 2.75–4.6 V (sputtering rate was 0.03 nm s^−1^ for 1500 s). As indicated in Fig. 6 and Supplementary Fig. 24, the chemical-depth profiles of several species for cycled undoped SNCM reveal the complex multilayers on its CEI film^4,51^. It is noted that the organic species (e.g., C_2_F^−^) and P-compounds (e.g., ${{{{{{\rm{PO}}}}}}}_{3}^{-}$) mainly coming from the decomposition of solvents/salts in electrolyte incline to locate in the outer surface layer of the CEI film. Undoped SNCM is found to be unstable at the delithiated state of 4.6 V, the reactions with organic electrolytes lead to its surface structural degradation and generation of transition metal fluorides (e.g., ${{{{{{\rm{NiF}}}}}}}_{3}^{-}$ and ${{{{{{\rm{MnF}}}}}}}_{3}^{-}$) in the inner layer of its CEI film. However, the amount of C_2_F^−^, ${{{{{{\rm{PO}}}}}}}_{3}^{-}$, ${{{{{{\rm{NiF}}}}}}}_{3}^{-}$ and ${{{{{{\rm{MnF}}}}}}}_{3}^{-}$ species formed in AZ0.3-SNCM cathode CEI film are much lower than those for undoped SNCM (Fig. 6a–d and Supplementary Fig. 24a–d). The 3D render (Fig. 6e and Supplementary Fig. 24e) and TOF-SIMS chemical imaging (Fig. 6f and Supplementary Fig. 24f) were collected to visualize the concentration gradient of these degradation-induced chemical species after cycling. As indicated in the Fig. 6, the degradation-induced fragements on undoped SNCM and AZ0.3-SNCM surface can be further confirmed by the cumulative signals of C_2_F^−^/${{{{{{\rm{PO}}}}}}}_{3}^{-}$ and ${{{{{{\rm{NiF}}}}}}}_{3}^{-}$/${{{{{{\rm{MnF}}}}}}}_{3}^{-}$, respectively. These results are also evidenced by XPS testing results obtained on undoped SNCM and AZ0.3-SNCM surface (Supplementary Fig. 25), indicating that the partially aggregation of Zr in the outer surface layer of AZ0.3-SNCM can effectively suppress the cathode/electrolyte side reaction and alleviate the transition cations dissolution. Especially, the benefits of the trace amount of Al/Zr codoping to Ni stability in AZ0.3-SNCM are reaffirmed.

## Discussion

### Structural stability and doping mechanisms

**Fig. 7:**
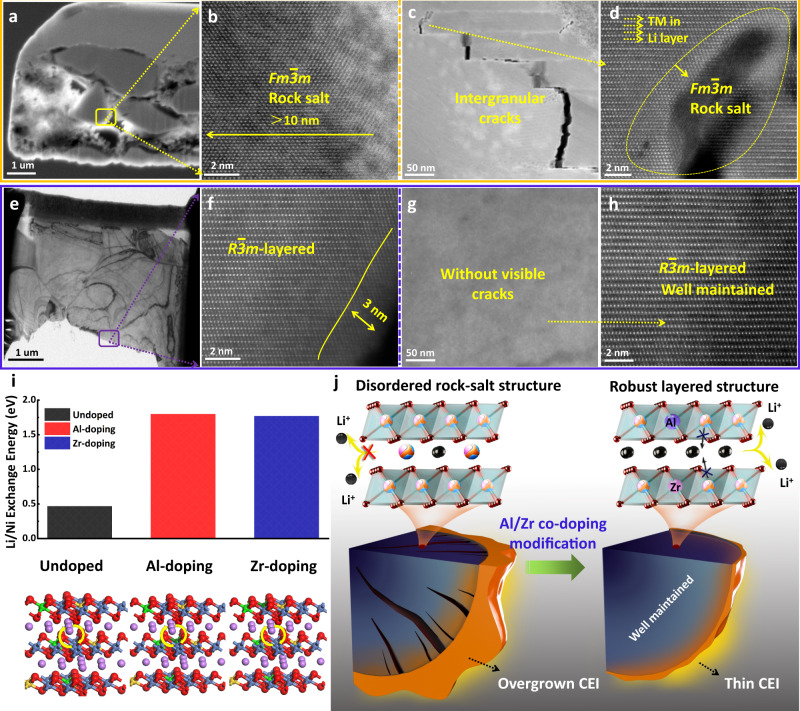
Morphology and crystal structure of undoped SNCM and AZ0.3-SNCM after 150 cycles at 100 mA g^−1^ within 2.75–4.6 V. **a**, **b** TEM and corresponding HAADF-STEM images of particle surface for undoped SNCM. **c**, **d** Cross-sectional TEM and corresponding STEM images of interior region for FIB-prepared undoped SNCM. **e**, **f** TEM and corresponding HAADF-STEM images of particle surface for AZ0.3-SNCM. **g**, **h** Cross-sectional TEM and corresponding STEM images of interior region for FIB-prepared AZ0.3-SNCM. **i** Li/Ni exchange energy of undoped SNCM, Al doping and Zr doping SNCM. **j** Schematic illustration of crack evolution and the internal morphological difference for undoped SNCM and AZ0.3-SNCM electrodes during cycling.

The morphological and structural stabilities are further investigated by cross-sectional SEM and TEM. As shown in Fig. 7a and Supplementary Fig. 26, many obvious nanocracks are observed within undoped SNCM particles after 150 cycles at 0.5 C (100 mA g^−1^) within 2.75–4.6 V. The high resolution scanning transmission electron microscopy (STEM) image (Fig. 7b) shows that a reconstruction layer of rock-salt phase (Fm$\bar{3}$m) with a thickness >10 nm presents on the outer surface of undoped SNCM, indicating the occurance of severe surface parastic reactions accompanied by undesirable phase transition. More importantly, as displayed in the cross-sectional TEM image prepared by focused ion beam (FIB), the irreversible phase transition is observed to extend along the crack to the inside of the particle (Fig. 7c), which further exacerbate structural and electrochemical degradations (Fig. 7d). The accumulation of internal-strain in undoped SNCM during cycling at the high charging cut-off voltage of 4.6 V is mainly ascribed to responsible for the generation of nanocracks^52–54^, and it will impede the Li^+^ diffusion in the sample and cause the structure collapse.

In contrast, AZ0.3-SNCM displays a smooth surface without any nanocracks and can remain its mechanical integrity after 150 cycles at 0.5 C (100 mA g^−1^) within 2.75–4.6 V (Supplementary Fig. 27). Moreover, AZ0.3-SNCM can maintain its original layered structure (R$\bar{3}$m) and only about 3 nm thin layer of disordered phase is detected on the particle surface (Fig. 7e, f). Moreover, nanocrack is absent from AZ0.3-SNCM single crystals (Fig. 7g), demonstrating the boosted structural stability during cycling at the high cut-off voltage of 4.6 V. Figure 7h illustrates a uniform atomic contrast along with transition metal layers, similar to the pristine AZ0.3-SNCM (Fig. 1c), which suggests that the irreversible phase transition is effectively suppressed in AZ0.3-SNCM. Additionally, as presented in the XRD pattern of cycled electrodes (Supplementary Fig. 28), the AZ0.3-SNCM exhibits a robust structural stability with well-maintained crystallinity, confirmed by the fixed position of (003) peak, which represent the initial layered hexagonal (R$\bar{3}$m) structure. Moreover, according to the analysis of the EIS measurements (Supplementary Fig. 18b) by using the inset equivalent circuit model, it shows an order of magnitude higher Li^+^ diffusion mobility for the AZ0.3 SNCM compared to the undoped SNCM.

The above results indicate that trace amount of Al/Zr co-doping in AZ0.3-SNCM can achieve an enhanced rate capability and cycling stability at the high charging cut-off voltage of 4.6 V. Single-crystalline architecture and Al/Zr co-doping in SNCM have multiple advantages. First, undoped SNCM and AZ0.3-SNCM single crystals are not subjected to the intragranular cracks, the structural degradation caused by the cathode/electrolyte side reactions along the intergranular cracks are effectively inhibited. Second, the incorporation of trace amount of Al/Zr in SNCM can enhance the Li^+^ ions diffusion mobility, resulting an improved electrode reaction kinetics and rate performance. Interestingly, we find that the doped Al ions with high solubility in SNCM are well incorporated its parent lattice, the high-valence Zr ions have limited solubility in SNCM, and part of them incline to aggregate on the outer surface layer. The incorporated Al/Zr dopants in bulk AZ0.3-SNCM can serve as pillars to reduce the *c*-axis contraction and release the internal-strain during cycling, which can effectively stabilize the SNCM crystal structure. Meanwhile, it can also enhance covalency of Ni^4+^–O^2−^ bonds and prevent the reduction of highly oxidized Ni^4+^ ions into Ni^2+^ ions and migration of Ni^2+^ ions into Li-layer with reduce Li/Ni cation mixing. The benefits of trace Al/Zr co-doping to mitigate the Li/Ni cation mixing can also be verified by the DFT calculation results. Whether pristine state (Fig. 7i) or delithiated state (Supplementary Fig. 29), the Li/Ni exchange energy of Zr-doped and Al-doped SNCM are higher than that of bare SNCM. It demonstrates that the energy barrier for Ni^2+^ ions migration to Li-layer is difficult in AZ0.3-LNCM compared with undoped SNCM. Furthermore, the aggregation of Zr on the outer surface layer of AZ0.3-SNCM can effectively prevent the dissolution of transition metals from AZ0.3-SNCM and inhibit undesirable phase transition induced by the electrolyte. Overall, as illustrated in Fig. 7j, the present study indicates that AZ0.3-SNCM is a promising cathode material for Li-based batteries, and this work presents a method to develop Ni-rich NCM cathode materials at a high charging cut-off voltage.

In summary, trace amount of Al/Zr in situ co-doped single-crystalline LiNi_0.88_Co_0.09_Mn_0.03_O_2_ Ni-rich cathode materials have been fabricated by the combination of co-precipitation and calcination method. The concurrent incorporation of Al/Zr in SNCM plays a dominant role in enhancing its Li^+^ ions diffusion mobility and serves as pillars to reduce its *c*-axis contraction at high delithiated state, which improves electrochemical performance, and suppresses particle cracking as well as structure degradation. While, the aggregation of Zr on outer surface layer of SNCM can effectively mitigate the undersired cathode-electrolyte interfacial reactions with alleviated transition metal cations dissolution. These results demonstrate that the functional trace doping of dual-elements, e.g., Al and Zr, is an effective strategy to address the issues of Ni-rich single crystal, including the internal strain accumulation, structural/mechanical degradation, and surface parasitic reaction, which is beneficial to achieve satisfactory electrochemical performance and accelerate the wide commercial application of Ni-rich NCM single crystals.

## Methods

### Materials synthesis

The spherical precursors of AZ0.3-SNCM samples were synthesized via a co-precipitation method. Stoichiometric amount of NiSO_4_·6H_2_O, CoSO_4_·7H_2_O, and MnSO_4_·5H_2_O (all are 2 mol L^−1^) were dissolved in deionized (DI) water to obtain a homogeneously mixed solution, and then pumped into the reaction tank. Then the NaOH (5 mol L^−1^, used as the precipitation agent and controlled by the pH meter) and NH_3_·H_2_O (4 mol L^−1^, used as chelating agent) solutions were separately pumped into the continuously stirred tank reactor (CSTR, 200 L) under N_2_ atmosphere. Particularly, the Al-precursor Al_2_(SO_4_)_3_·6H_2_O (0.5 mol L^−1^) was pumped into the tank reactor accompanied with the main materials (NiSO_4_, CoSO_4_, and MnSO_4_ solutions) due to its relatively higher solubility. However, the Zr-precursor Zr(SO_4_)_2_·4H_2_O (0.5 mol L^−1^) was pumped into the tank reactor in the middle of the entire reaction process owing to its poor solubility. The Ni_0.874_Co_0.09_Mn_0.03_Al_0.003_Zr_0.003_(OH)_2_ precursors were finally obtained through filtering the precipitations, washing, and drying in a vacuum oven at 110 °C overnight. Al ions with high solubility in SNCM are well incorporated its parent lattice. The precursors were thoroughly mixed with LiOH·H_2_O (Li: M ratio = 1.06:1) and calcined at 820 °C for 10 h in oxygen atmosphere. Due to their trace doping concentration, Al/Zr ions were well incorporated their parent lattice to obtain AZ0.3-SNCM cathode during the high-temperature sintering process. Therefore, the Zr-atoms are fully located at outer surface layer, while Al-atoms incline to homogeneous dispersed among the bulk, eventually leading to the higher Zr doping concentration on the outer surface layer and uniform Al doping concentration in the bulk. For comparison, the pristine and Al or/and Zr-doped SNCM single-crystalline samples were fabricated by a similar method.

### Materials characterizations

The chemical compositions of the samples were identified by ICP (OPIMA 8300, Perkin Elmer). Crystal structure of the samples was determined by using a Rint-2000, Rigaku type X-ray diffractometer within 5°–120° at a scan rate of 2° min^−1^. The collected XRD data were analyzed by using the Rietveld refinement program (General Structure Analysis System, GASA) software package. For in situ XRD testing, a specially designed Swagelok cell equipped with X-ray-transparent aluminum window was used for in situ measurements. The Li metal (China Energy Lithium Co., Ltd, thickness of 0.3 mm, purity of 99.9%) and the electrolyte of 1 M LiPF_6_ in ethyl carbonate/diethylene carbonate (EC/DEC, 1:1 in volume, H_2_O < 6.9 ppm) were used as the counter electrode and electrolyte, respectively. The in situ XRD patterns were collected with an interval of 40 min for each 2θ scan from 10 to 60° with 0.02° step increment during the charging/discharging process at a current rate of 0.1 C (1 C = 200 mA g^−1^ considering the mass of the positive electrode active material for in situ XRD measurements).

Morphology and microstructure of the samples were studied by utilizing the scanning electron microscopy (SEM, JSM 6400, JEOL) equipped with an energy dispersive spectrometer (EDS), transmission electron microscopy (TEM, JEOL 2100 F, JEOL), and the spherical aberration-corrected transmission electron microscopy (ACTEM, FEI Titan G2 80–200 ChemiSTEM). Besides, the samples were pretreated by focused ion beam (FIB, SCIOS, FEI) before TEM and ACTEM tests. X-ray photoelectron spectroscopy (XPS) was conducted by using the Thermo Fisher ESCALAB 250Xi. TOF-SIMS was applied on ION-TOF GmbH TOF SIMS 5–100, with the sputtering rate of 0.03 nm s^−1^, mass resolution of >12,000, and depth resolution of <1 nm. While, the area of typical sputtering on cycled SNCM electrodes was 150 × 150 µm. The X-ray absorption spectra were collected on the beamline BL01C1 in NSRRC. The radiation was monochromatized by a Si (111) double-crystal monochromator. XANES and EXAFS data reduction and analysis were processed by Athena software.

Furthermore, 3D synchrotron X-ray fluorescence mapping was performed at the HXN beamline of NSLS II at Brookhaven National Laboratory. The nanoprobe experiment was applied at 12 keV by focusing the coherent monochromatic X-rays down to a 12 nm spot size using a pair of multilayer Laue lenses38. Tomography measurements were conducted by collecting a total of 91 projections from −90° to 90°, with 2° intervals.

### Electrochemical measurements

Electrochemical performances of as-prepared cathode materials were assessed using CR2032 coin-type half-cell. The active materials (89 wt%), conductive super P (5 wt%) and KS-6 (3.5 wt%) additive, and binder polyvinylidene fluoride (PVDF) (2.5 wt%) were dissolved in N-methyl-1,2-pyrrolidone solvent (NMP) to obtain the homogeneous cathode slurry by using a mortar in a dry room (the Dew point is −45 and the size of the room is 15 m^2^). The cathode slurry was then coated on the Al foil current collector (Huizhou Xinfeile New Material Co., Ltd, thickness of 12 μm, purity of 99.3%) and casted into a ϕ12 mm tablet to form the cathode electrode with a mass loading of 8.5 ± 0.15 mg cm^−2^. The cells were assembled in an argon-filled glovebox (both the water and oxygen contents are less than 0.1 ppm) with Li metal (China Energy Lithium Co., Ltd, thickness of 0.3 mm, purity of 99.9%) and 1 M LiPF_6_ in ethyl carbonate/diethylene carbonate (EC/DEC, 1:1 in volume, H_2_O < 6.9 ppm) as the counter electrode and the electrolyte solution, respectively.

For pouch-type full cells, the cathode electrodes were prepared by a mixture slurry of active material (94 wt%), carbon black (2 wt%), KS-6 (2 %), and PVDF (2 wt%). 1.1 M LiPF6 in ethyl carbonate/ethyl methyl carbonate/diethyl carbonate (EC/EMC/DEC, 3:5:2 in volume, H_2_O < 6.9 ppm) + 1 wt% vinylene carbonate was used as electrolyte. When the artificial graphite (99.7%, Shenzhen Kejing Star Technology Co., Ltd, average size of 20 μm) was used as an anode, which was casted on the Cu foil current collector (Guangdong Jiayuan Technology Co., Ltd, thickness of 8 μm, purity of 99.8%). The loading mass of cathode and anode (graphite) was around 31 mg cm^−2^ (ca. 6.51 mAh cm^−2^) and 21.4 mg cm^−2^ (ca. 7.28 mAh cm^−2^) on both sides, respectively. The designed capacities of anode and cathode were 340 and 210 mAh g^−1^, respectively. The pouch cells were assembled with winding process. The capacity balance of anode to cathode was approximately 1.13, while the cells capacity was about 1.85 Ah. When Li-metal belt (China Energy Lithium Co., Ltd, thickness of 100 μm, purity of 99.5%) was applied as anode material, the mass loading of cathode is 60 mg cm^−2^ (ca. 12.6 mAh cm^−2^) on both sides. The pouch cells were assembled with stacked process, and the cells capacity is designed about 10.83 Ah or 2.04 Ah. For electrode sample’s preparation for ex situ measurements, these cycled electrodes from coin-type cells were washed by soaking in the dimethyl carbonate (Aladdin Co. Ltd, ≥99.9 wt%, H_2_O < 10 ppm) solution for 1 h, cut and collected in Ar-atmospheres under inert atmospheres, while the sample transfer bin with an inert atmosphere was used to avoid air contamination.

Galvanostatic charge/discharge tests were conducted between 2.75 and 4.3 V (or 4.4/4.6 V) at 25 ± 2 °C or 55 ± 2 °C (Land CT2001A for coin-type cell, Neware CT-4000 for pouch-type cell) in the constant environmental chamber. For the current rate, the grams refer to the cathode active material for all coin-type half-cell and pouch-type full-cell measurements. A CHI660E electrochemical workstation was applied to characterize the cyclic voltammetry (CV, sweep rate: 0.1 mV s^−1^, voltage window: 3.2–4.6 V), while Bio-Logic EC-LAB SP-300 electrochemical workstation was used to collect the electrochemical impedance spectroscopy (EIS, frequency range: 100,000–0.01 Hz, 10 data points per decade of frequency, signal amplitude: 5 mV), which was operated at open-circuit voltage before cycling and quasi-stationary potential of 2.75 V with fully discharging after cycling.

### Calculation methods

The DFT calculations were performance with the exchange-correlation functional of Perdew-Burke-Ernzerhof (PBE) by Vienna ab-initio simulation package (VASP)^55,56^. To descript the strong correlation effect of d orbital in V, the Hubbard U (the effective U of 6.7, 4.2, and 4.9 eV for Ni, Mn, and Co, respectively) was employed^42^. The plane-wave energy cutoff was set to be 400 eV. The Fermi scheme was employed for electron occupancy with an energy smearing of 0.1 eV. The first Brillouin zone was sampled in the Monkhorst-Pack grid^57^. These calculations were performed with a 2×2×1 k-point mesh. The energy (converged to 1.0 × 10^−6^ eV/atom) and force (converged to 0.03 eV/Å) were set as the convergence criterion for geometry optimization. The spin polarization was considered in all calculations. The transition state (TS) structures and reaction pathways were located using the climbing image nudged elastic band (CI-NEB) method with four imgates^58^, and the minimum energy pathway was optimized until the maximum force was less than 0.05 eV/Å.

The layered ternary compound was simulated by using the periodic slab model of LiNiO_2_ with 3Mn and 3Co doped, each model containing 15Li, 21Ni, 3Mn, 3Co, and 54O atoms. The Mn, Co, and Ni would be replaced by Al and Zr to estimate the model Al and Zr doping, respectively. The formation energy (∆*E*_form_) of Al or Zr doping would be employed to estimate the stability, which is defined as: ∆*E*_form_ = *E*_slab-m_ − *E*_slab_ − *E*_m_, where the *E*_slab-m,_ *E*_slab_, and *E*_m_ denote the total energies of slab with metal m doped, the clean slab and metal atomic energy derived from metal bulk, respectively. The more negative value of ∆*E*_form_, the more stable doped configuration will be obtained.

### Reporting summary

Further information on research design is available in the Nature Research Reporting Summary linked to this article.

## Supplementary information

Supporting information

Reporting Summary

## Data Availability

All relevant data that support the findings of this study are presented in the manuscript and supplementary information file. Source data are available from the corresponding author upon reasonable request.

## References

[1] Cano ZP (2018). Batteries and fuel cells for emerging electric vehicle markets. Nat. Energy.

[2] Li M, Lu J (2020). Cobalt in lithium-ion batteries. Science.

[3] Zhang X-D (2020). Structure design of cathode electrodes for solid-state batteries: challenges and progress. Small Struct..

[4] Li W (2017). Dynamic behaviour of interphases and its implication on high-energy-density cathode materials in lithium-ion batteries. Nat. Commun..

[5] Bianchini M, Roca-Ayats M, Hartmann P, Brezesinski T, Janek J (2019). There and back again-the journey of LiNiO_2_ as a cathode active material. Angew. Chem. Int. Ed..

[6] Zeng X, Zhan C, Lu J, Amine K (2018). Stabilization of a high-capacity and high-power nickel-based cathode for Li-ion batteries. Chem.

[7] Kim J (2018). Prospect and reality of Ni-rich cathode for commercialization. Adv. Energy Mater..

[8] Yan P (2017). Intragranular cracking as a critical barrier for high-voltage usage of layer-structured cathode for lithium-ion batteries. Nat. Commun..

[9] Liu H (2017). Intergranular cracking as a major cause of long-term capacity fading of layered cathodes. Nano Lett..

[10] Liu G (2018). Single-Crystalline particles: An effective way to ameliorate the intragranular cracking, thermal stability, and capacity fading of the LiNi_0.6_Co_0.2_Mn_0.2_O_2_ electrodes. J. Electrochem. Soc..

[11] Nam GW (2019). Capacity fading of Ni-rich NCA cathodes: Effect of microcracking extent. ACS Energy Lett..

[12] Park K-J (2018). Improved cycling stability of Li[Ni_0.90_Co_0.05_Mn_0.05_]O_2_ through microstructure modification by boron doping for Li-ion batteries. Adv. Energy Mater..

[13] Sun H-H, Manthiram A (2017). Impact of microcrack generation and surface degradation on a nickel-rich layered Li[Ni_0.9_Co_0.05_Mn_0.05_]O_2_ cathode for lithium-ion batteries. Chem. Mater..

[14] Xu X (2019). Radially oriented single-crystal primary nanosheets enable ultrahigh rate and cycling properties of LiNi_0.8_Co_0.1_Mn_0.1_O_2_ cathode material for lithium-ion batteries. Adv. Energy Mater..

[15] Li H (2019). Synthesis of single crystal LiNi_0.88_Co_0.09_Al_0.03_O_2_ with a two-step lithiation method. J. Electrochem. Soc..

[16] Li H, Li J, Ma X, Dahn JR (2018). Synthesis of single crystal LiNi_0.6_Mn_0.2_Co_0.2_O_2_ with enhanced electrochemical performance for lithium ion batteries. J. Electrochem. Soc..

[17] Bi Y (2020). Reversible planar gliding and microcracking in a single-crystalline Ni-rich cathode. Science.

[18] Cha H (2020). Boosting reaction homogeneity in high-energy lithium-ion battery cathode materials. Adv. Mater..

[19] Fan X (2020). Unravelling the influence of quasi single-crystalline architecture on high-voltage and thermal stability of LiNi_0.5_Co_0.2_Mn_0.3_O_2_ cathode for lithium-ion batteries. Chem. Eng. J..

[20] Liu T (2021). Understanding Co roles towards developing Co-free Ni-rich cathodes for rechargeable batteries. Nat. Energy.

[21] Zhang Y (2019). LiNi_0.90_Co_0.07_Mg_0.03_O_2_ cathode materials with Mg-concentration gradient for rechargeable lithium-ion batteries. J. Mater. Chem. A.

[22] Huang Y (2019). Tellurium surface doping to enhance the structural stability and electrochemical performance of layered Ni-rich cathodes. ACS Appl. Mater. Interfaces.

[23] Mu L (2019). Dopant distribution in Co-free high-energy layered cathode materials. Chem. Mater..

[24] Liu T (2021). Rational design of mechanically robust Ni-rich cathode materials via concentration gradient strategy. Nat. Commun..

[25] Maleki Kheimeh Sari H, Li X (2019). Controllable cathode-electrolyte interface of Li[Ni_0.8_Co_0.1_Mn_0.1_]O_2_ for lithium ion batteries: A review. Adv. Energy Mater..

[26] Liang C (2022). Insight into the structural evolution and thermal behavior of LiNi_0.8_Co_0.1_Mn_0.1_O_2_ cathode under deep charge. J. Energy Chem..

[27] Feng Z (2021). Dual-Element-Modified single-crystal LiNi_0.6_Co_0.2_Mn_0.2_O_2_ as a highly stable cathode for lithium-ion batteries. ACS Appl Mater. Interfaces.

[28] Fan X (2020). Crack-free single-crystalline Ni-rich layered NCM cathode enable superior cycling performance of lithium-ion batteries. Nano Energy.

[29] Schipper F (2016). Stabilizing nickel-rich layered cathode materials by a high-charge cation doping strategy: zirconium-doped LiNi_0.6_Co_0.2_Mn_0.2_O_2_. J. Mater. Chem. A.

[30] Cheng X (2019). Realizing superior cycling stability of Ni-Rich layered cathode by combination of grain boundary engineering and surface coating. Nano Energy.

[31] Liao JY, Oh SM, Manthiram A (2016). Core/double-shell type gradient Ni-rich LiNi_0.76_Co_0.10_Mn_0.14_O_2_ with high capacity and long cycle life for lithium-ion batteries. ACS Appl. Mater. Interfaces.

[32] Yang H (2019). Simultaneously dual modification of Ni‐rich layered oxide cathode for high‐energy lithium‐ion batteries. Adv. Funct. Mater..

[33] Oh P (2016). High-Performance heterostructured cathodes for lithium-ion batteries with a Ni-rich layered oxide core and a Li-rich layered oxide shell. Adv. Sci..

[34] Sun Y-Y, Liu S, Hou Y-K, Li G-R, Gao X-P (2019). In situ surface modification to stabilize Ni-rich layered oxide cathode with functional electrolyte. J. Power Sources.

[35] Beltrop K (2018). Triphenylphosphine oxide as highly effective electrolyte additive for graphite/NMC811 lithium ion cells. Chem. Mater..

[36] Kim U-H, Kuo L-Y, Kaghazchi P, Yoon CS, Sun Y-K (2019). Quaternary layered Ni-rich NCMA cathode for lithium-ion batteries. ACS Energy Lett..

[37] Liu Y (2019). Enhancement on structural stability of Ni-rich cathode materials by in-situ fabricating dual-modified layer for lithium-ion batteries. Nano Energy.

[38] Tamirat AG (2021). Ultrathin silicon nanolayer implanted Ni_x_Si/Ni nanoparticles as superlong-cycle lithium-ion anode material. Small Struct..

[39] Li HH (2007). Changes in the cation ordering of layered O3 Li_*x*_Ni_0.5_Mn_0.5_O_2_ during electrochemical cycling to high voltages: an electron diffraction study. Chem. Mater..

[40] Croguennec L, Pouillerie C, Mansour AN, Delmas C (2001). Structural characterisation of the highly deintercalated Li_*x*_Ni_1.02_O_2_ phases (with *x* ≤ 0.30). J. Mater. Chem..

[41] Min K, Seo SW, Song YY, Lee HS, Cho E (2017). A first-principles study of the preventive effects of Al and Mg doping on the degradation in LiNi_0.8_Co_0.1_Mn_0.1_O_2_ cathode materials. Phys. Chem. Chem. Phys..

[42] Zhang MJ (2018). Cationic ordering coupled to reconstruction of basic building units during synthesis of high-Ni layered oxides. J. Am. Chem. Soc..

[43] Lin F (2014). Surface reconstruction and chemical evolution of stoichiometric layered cathode materials for lithium-ion batteries. Nat. Commun..

[44] Yoon W-S (2005). Investigation of the charge compensation mechanism on the electrochemically Li-ion deintercalated Li_1−*x*_Co_1/3_Ni_1/3_Mn_1/3_O_2_ electrode system by combination of soft and hard X-ray absorption spectroscopy. J. Am. Chem. Soc..

[45] Deng T (2019). Designing in-situ-formed interphases enables highly reversible cobalt-free LiNiO_2_ cathode for Li-ion and Li-metal batteries. Joule.

[46] Xu J (2016). Understanding the degradation mechanism of lithium nickel oxide cathode for Li-ion batteries. ACS Appl Mater. Interfaces.

[47] Ding Z (2017). Understanding the enhanced kinetics of gradient-chemical-doped lithium-rich cathode material. ACS Appl. Mater. Interfaces.

[48] Zhang J (2020). A novel NASICON‐type Na_4_MnCr(PO_4_)_3_ demonstrating the energy density record of phosphate cathodes for sodium‐ion batteries. Adv. Mater..

[49] Zhang F (2020). Surface regulation enables high stability of single-crystal lithium-ion cathodes at high voltage. Nat. Commun..

[50] Zhang K (2019). Manganese based layered oxides with modulated electronic and thermodynamic properties for sodium ion batteries. Nat. Commun..

[51] Chen Y (2020). Armoring LiNi_1/3_Co_1/3_Mn_1/3_O_2_ cathode with reliable fluorinated organic–inorganic hybrid interphase layer toward durable high rate battery. Adv. Funct. Mater..

[52] Tian C (2018). Charge heterogeneity and surface chemistry in polycrystalline cathode materials. Joule.

[53] Fan X (2021). In situ inorganic conductive network formation in high-voltage single-crystal Ni-rich cathodes. Nat. Commun..

[54] Choi J (2019). The role of Zr doping in Li[Ni_0.6_Co_0.2_Mn_0.2_]O_2_ as a stable cathode material for lithium ion batteries. ChemSusChem.

[55] Perdew JP, Burke K, Ernzerhof M (1996). Generalized gradient approximation made simple. Phys. Rev. Lett..

[56] Kresse G, Furthmüller J (1996). Efficient iterative schemes for ab initio total-energy calculations using a plane-wave basis set. Phys. Rev. B.

[57] Monkhorst HJ, Pack JD (1976). Special points for Brillouin-zone integrations. Phys. Rev. B.

[58] Henkelman G, Uberuaga BP, Jónsson H (2000). A climbing image nudged elastic band method for finding saddle points and minimum energy paths. J. Chem. Phys..

