# Supplementary material for: Enabling high energy lithium metal batteries via single-crystal Ni-rich cathode material co-doping strategy
Source: Nat Commun. 2022 Apr 28;13:2319. doi: 10.1038/s41467-022-30020-4 (PMC9050889; doi:10.1038/s41467-022-30020-4)
Supplement: Supplementary file 1 — Supporting information [file 41467_2022_30020_MOESM1_ESM.pdf]

Supplementary Information

Enabling high energy lithium metal batteries via single-crystal Ni-rich cathode material co-doping strategy

Xing Ou[1,2,7], Tongchao Liu[3,7], Wentao Zhong[1], Xinming Fan[2,], Xueyi Guo[2], Xiaojing Huang[4], Liang Cao[1,2], Junhua Hu[5], Bao Zhang[2], Yong S. Chu[4], Guorong Hu[2], Zhang Lin[2], Mouad Dahbi[6], Jones Alami[6], Khalil Amine[3,*], Chenghao Yang[1,*], Jun Lu[3,*]*

[1]*Guangzhou Key Laboratory for Surface Chemistry of Energy Materials, New Energy Research Institute, School of Environment and Energy, South China University of Technology, Guangzhou 510006, China*

[2]*School of Metallurgy and Environment, Central South University, Changsha 410083, China*

[3]*Chemical Sciences and Engineering Division, Argonne National Laboratory, Lemont, Illinois 60439, USA*

[4]*National Synchrotron Light source II, Brookhaven National Laboratory, Upton, NY 11973, USA*

[5]*School of Materials Science and Engineering, Zhengzhou University, Zhengzhou 450001, China*

[6]*Materials Science and Nano-Engineering Department, Mohammed VI Polytechnic University, Ben Guerir, Morocco*

[7]*These authors contributed equally to this work.*

**Corresponding author: junlu@anl.gov (J.L.); fanxinming@csu.edu.cn (X.F.); esyangc@scut.edu.cn (C.Y.); and amine@anl.gov*

1. **Supplementary Figure 1-29**

2. **Supplementary Table 1-5**

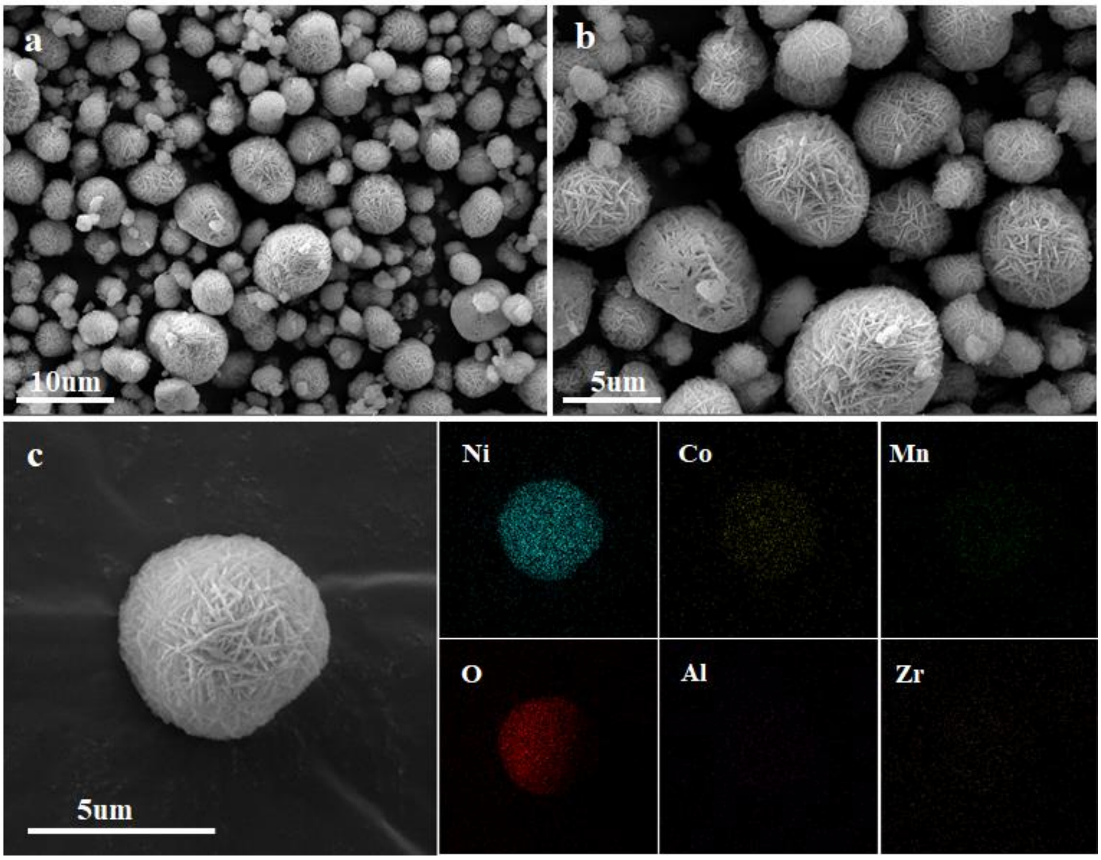

Supplementary Figure 1. SEM images (a, b) and EDS elemental mapping of Ni, Co, Mn, O, Al and Zr (c) for the $Ni_{0.874}Co_{0.09}Mn_{0.03}Al_{0.003}Zr_{0.003}(OH)_2$ precursors of AZ0.3-SNCM.

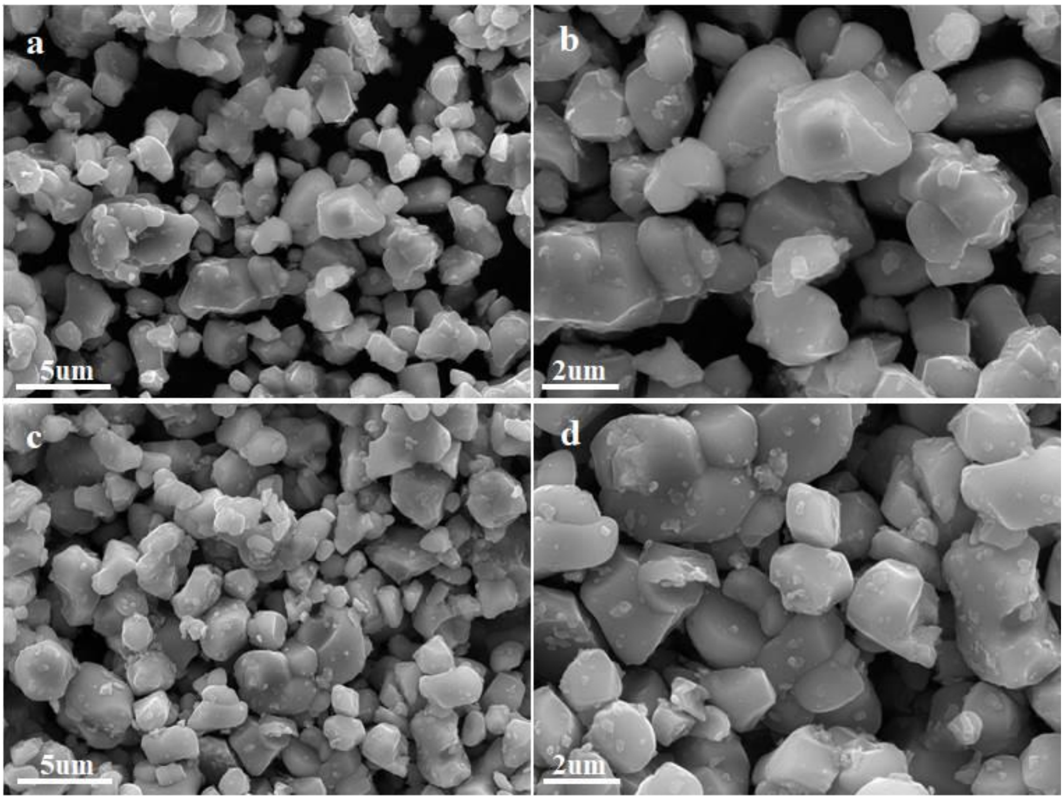

Supplementary Figure 2. SEM images of undoped SNCM (a, b) and AZ0.3-SNCM (c, d) cathode materials.

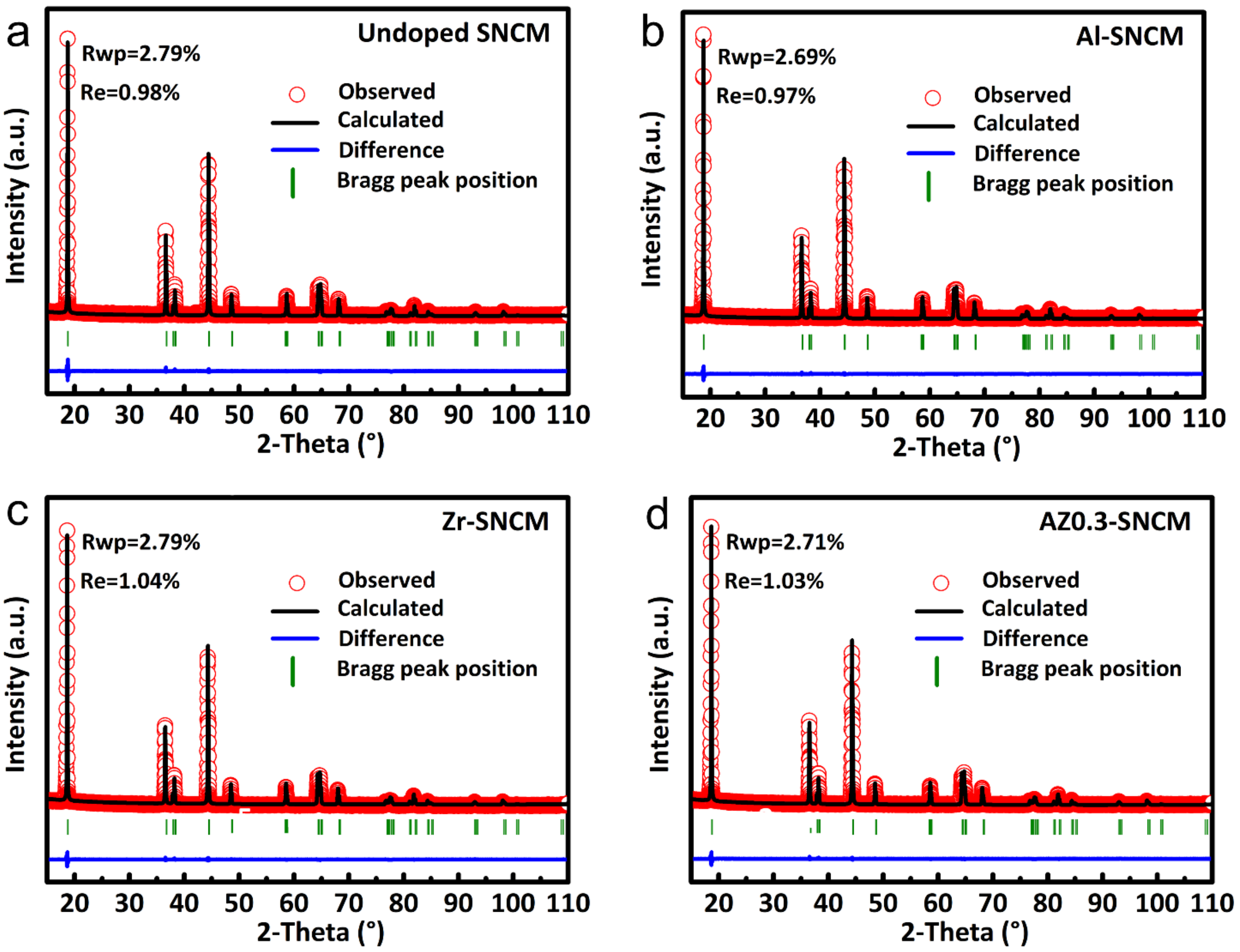

Supplementary Figure 3. Rietveld refinements of X-ray diffraction pattern for undoped SNCM (a), Al-SNCM (b), Zr-SNCM (c) and AZ0.3-SNCM (d).

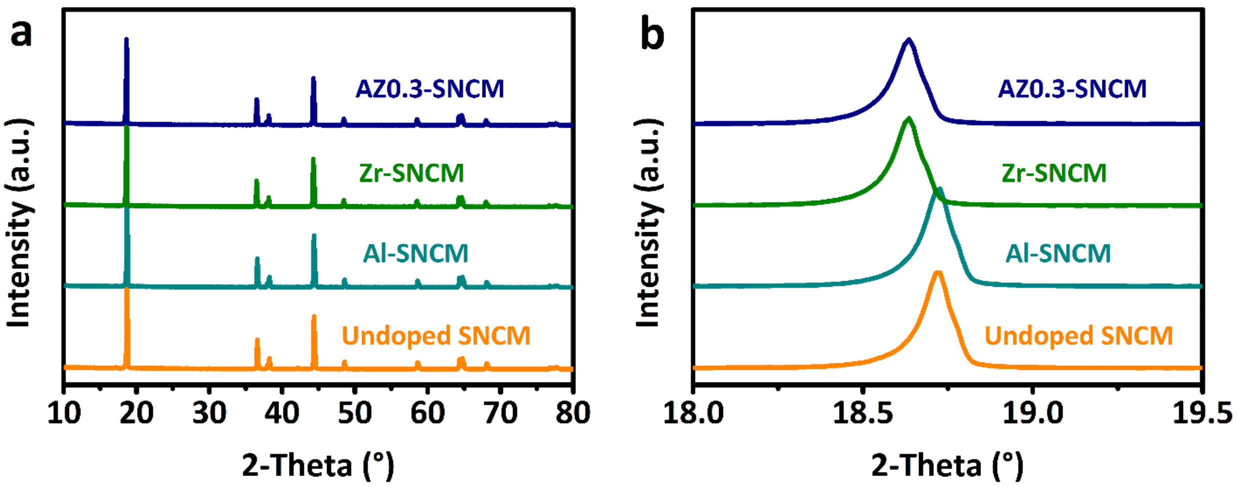

Supplementary Figure 4. X-ray diffraction (XRD) patterns for undoped SNCM, Al-SNCM, Zr-SNCM and AZ0.3-SNCM (a), and the enlarged view in the 2θ range of 18°-19.5° (b).

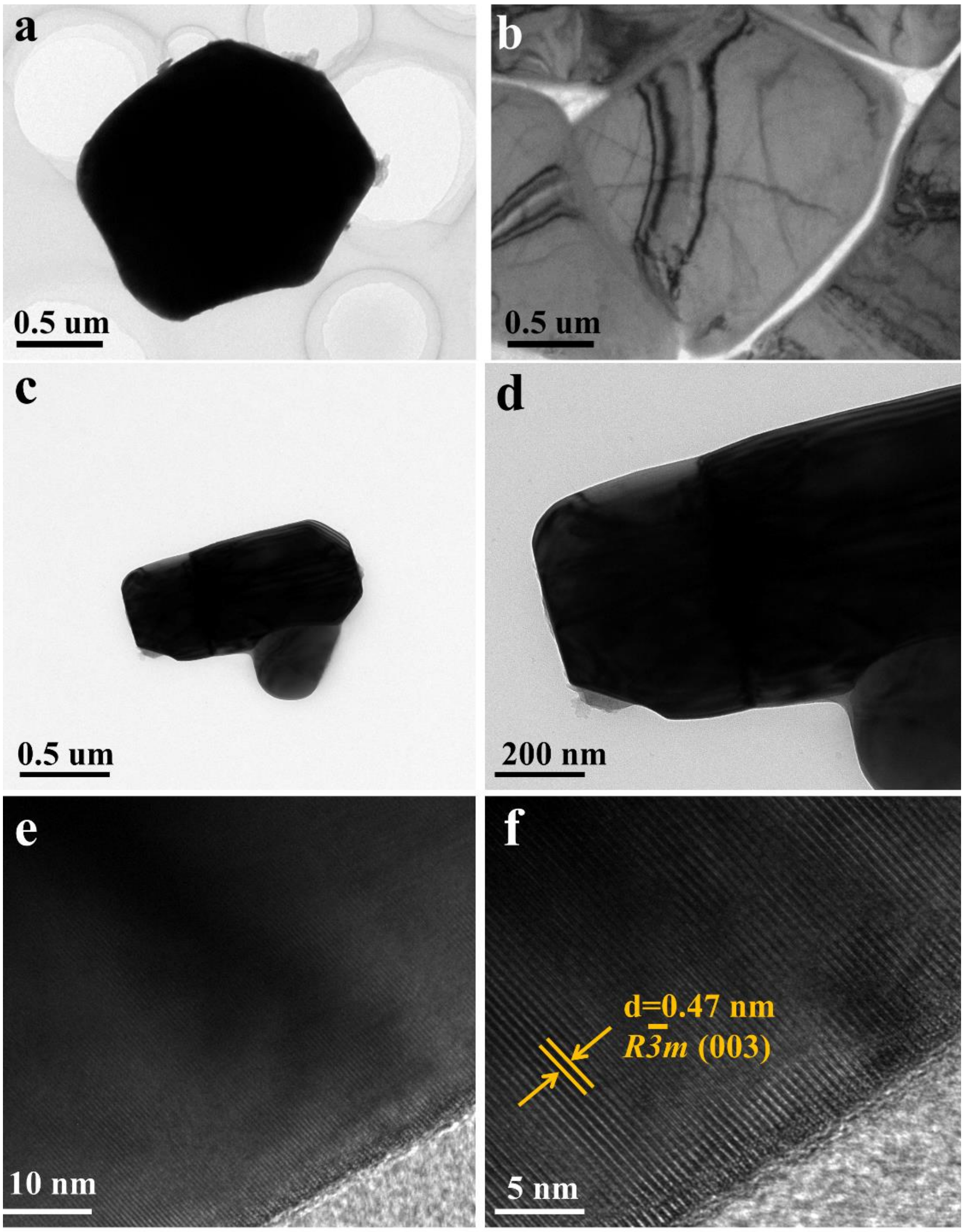

Supplementary Figure 5. TEM (a-d) and magnified HRTEM (e,f) images of AZ0.3-SNCM prepared by FIB treatment.

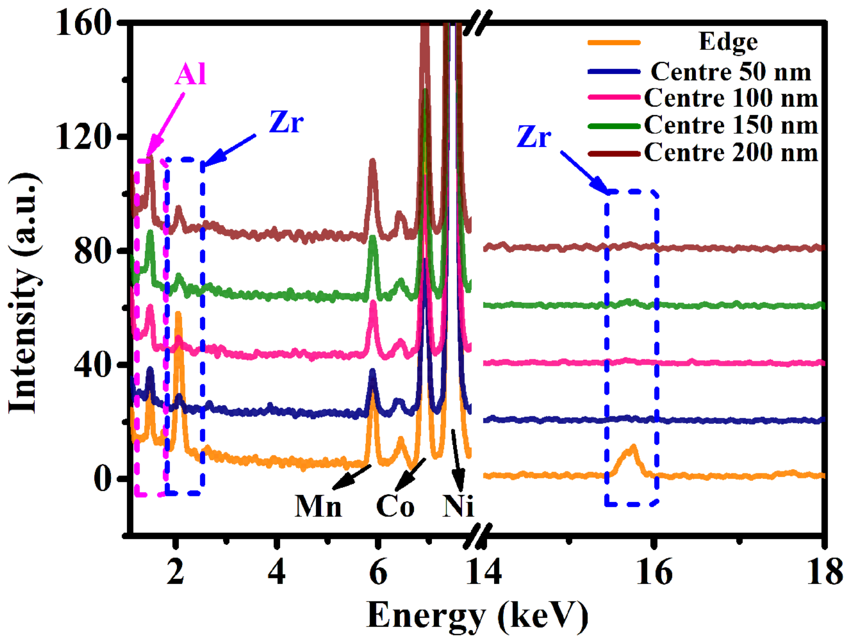

Supplementary Figure 6. In-depth EDS spectra of the selected area for AZ0.3-SNCM sample.

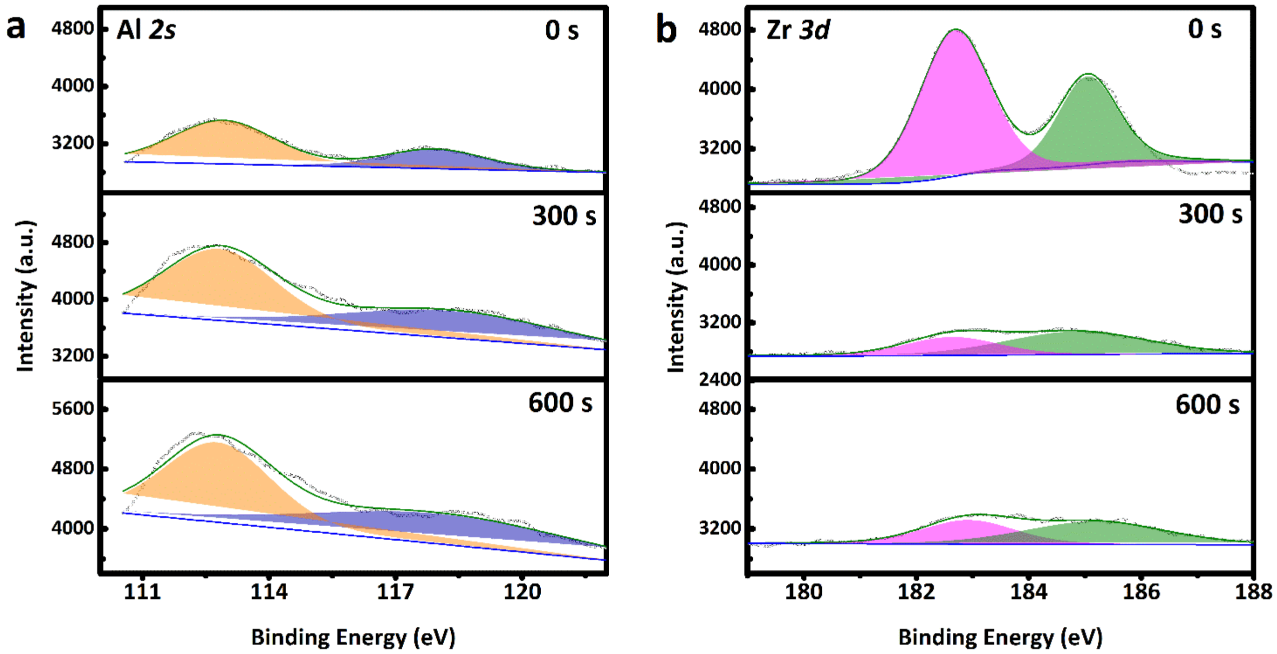

Supplementary Figure 7. In-depth XPS spectra of Al *2s* (a) and Zr *3d* (b) for AZ0.3-SNCM sample.

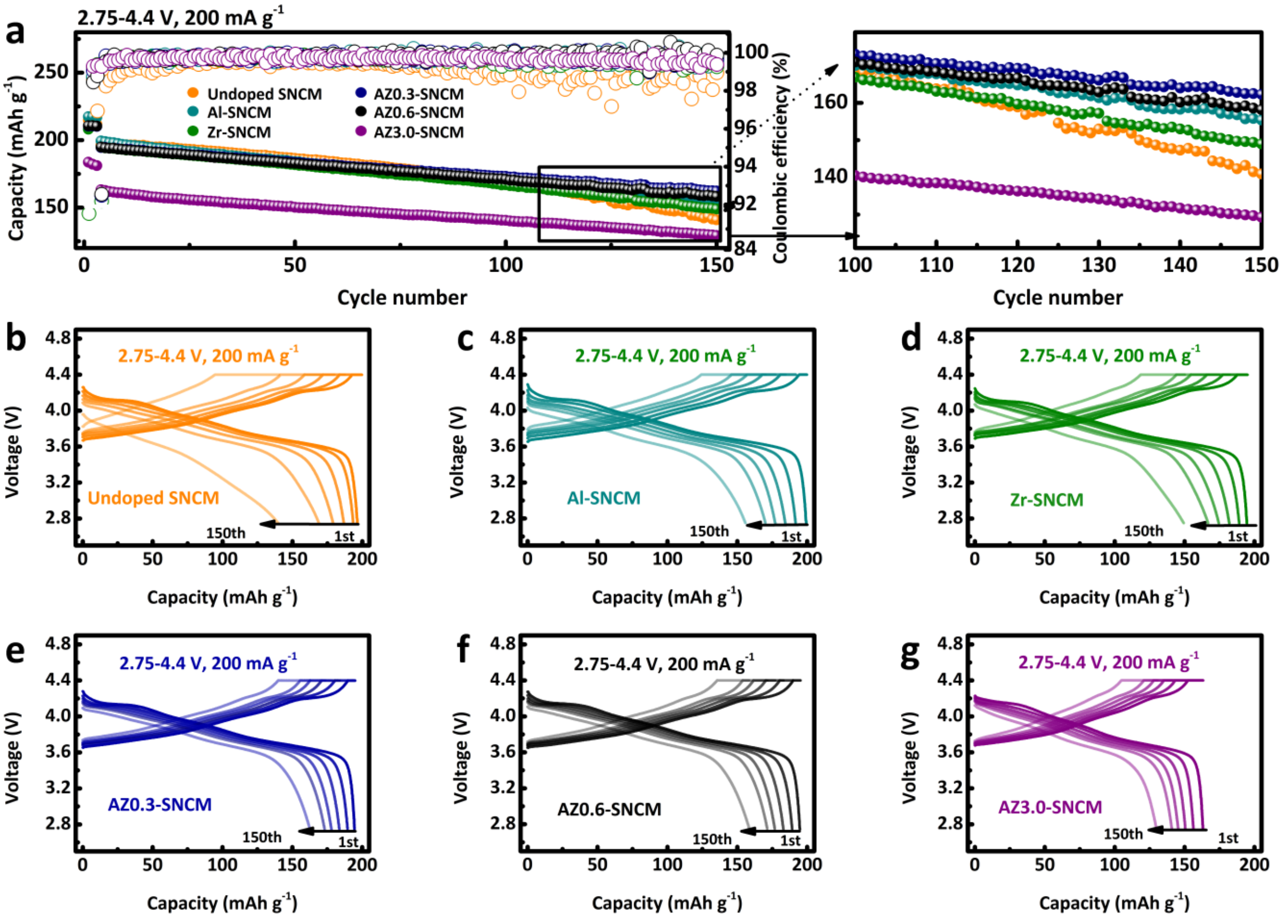

Supplementary Figure 8. Cycling performance tested by coin-type half-cell at 25 °C (Li metal anode and 1 M $LiPF_6$ in EC/DEC (1:1 in volume) are used as the counter electrode and electrolyte, respectively) for all samples (a) and charge/discharge curves for undoped SNCM (b), Al-SNCM (c), Zr-SNCM (d), AZ0.3-SNCM (e), AZ0.6-SNCM (f) and AZ3-SNCM (g) at 1 C (200 mA g^{-1}, the grams refer to the cathode active material for all coin-type half-cell measurements, similarly hereinafter) within the voltage range of 2.75-4.4 V at 25°C.

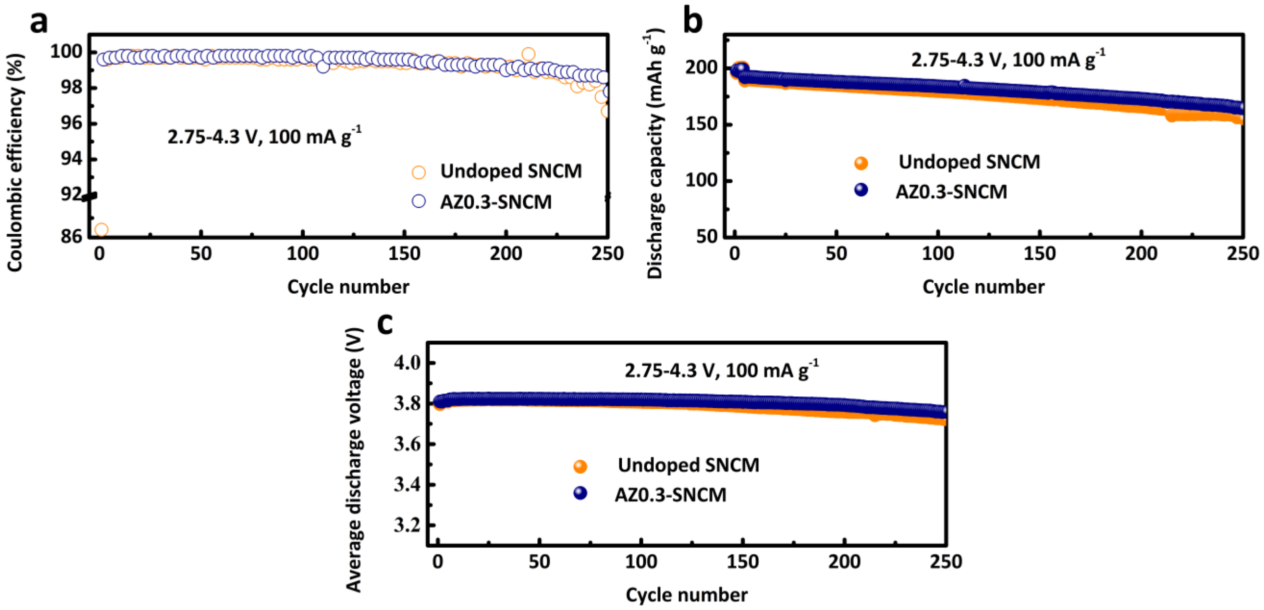

Supplementary Figure 9. Cycling performance tested by coin-type half-cell at 25 °C (Li metal anode and 1 M $LiPF_6$ in EC/DEC (1:1 in volume) are used as the counter electrode and electrolyte, respectively). Coulombic efficiency (a), discharge capacity (b) and average discharge voltage (c) for undoped SNCM and AZ0.3-SNCM samples during long-term cycling at 0.5 C (100 mA g^{-1}) within the voltage range of 2.75-4.3 V at 25°C.

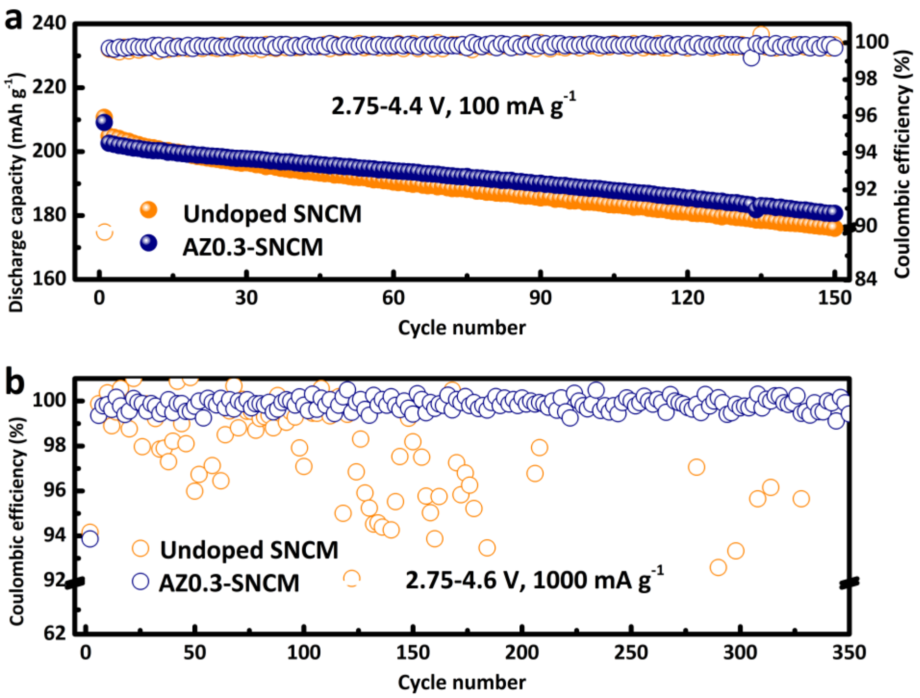

Supplementary Figure 10. Electrochemical performance tested by coin-type half-cell at 25 °C (Li metal anode and 1 M $LiPF_6$ in EC/DEC (1:1 in volume) are used as the counter electrode and electrolyte, respectively). Discharge capacity for undoped SNCM and AZ0.3-SNCM samples during long-term cycling at 0.5 C (100 mA g^{-1}) within the voltage range of 2.75-4.4 V at 25°C (a). Coulombic efficiency for undoped SNCM and AZ0.3-SNCM samples during long-term cycling at 5 C (1000 mA g^{-1}) within the voltage range of 2.75-4.6 V at 25°C (b).

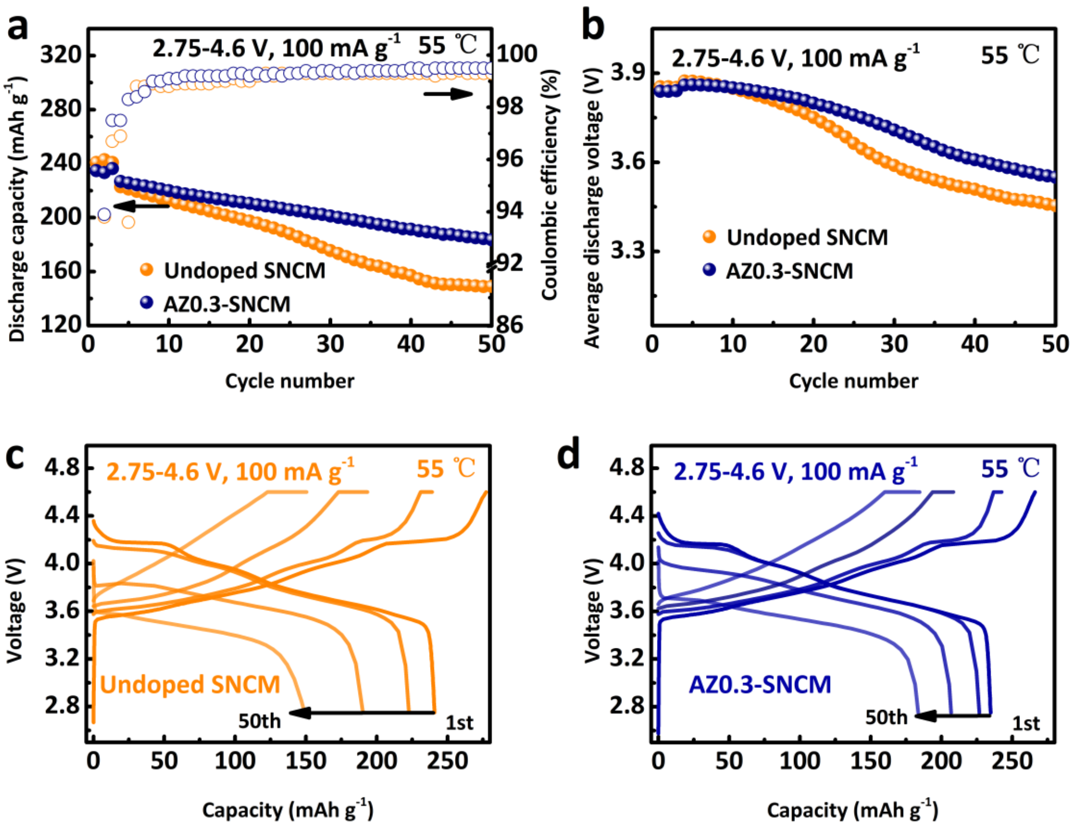

Supplementary Figure 11. Cycling performances tested by coin-type half-cell at 25 °C (Li metal anode and 1 M $LiPF_6$ in EC/DEC (1:1 in volume) are used as the counter electrode and electrolyte, respectively). for undoped SNCM (a) and AZ0.3-SNCM (b) cathodes. Charge/discharge curves at 0.5 C (100 mA g^{-1}) for undoped SNCM (c) and AZ0.3-SNCM (d) cathodes within the voltage range of 2.75-4.6 V at 55 °C.

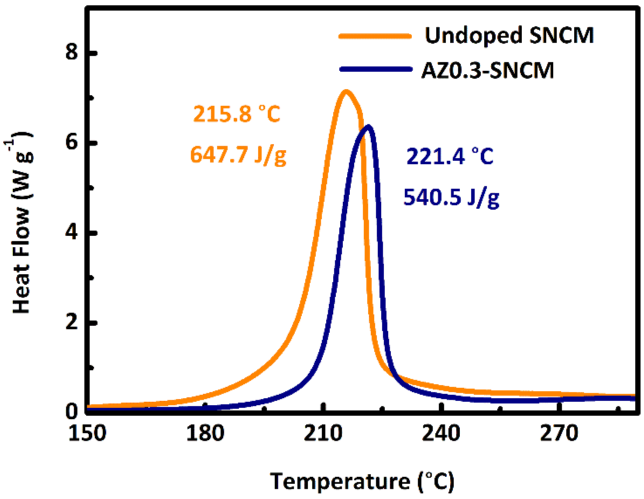

Supplementary Figure 12. DSC traces of the electrochemically delithiated undoped SNCM and AZ0.3-SNCM electrodes charged to 4.6 V.

To compare the thermal stability, both SNCM and AZ0.3-SNCM cathodes are charged to 4.6 V, disassembled from the coin-cells and reacted with electrolyte, and eventually analyzed by DSC in Ar atmosphere within temperature range from 25 to 300 ºC. It is noted that the exothermic peak of AZ0.3-SNCM cathode (221.4 ºC) is much higher with less heat release than undoped SNCM cathode, demonstrating better thermal stability and enhanced structural integrity of AZ0.3-SNCM, especially at the highly delithiated state of 4.6 V.

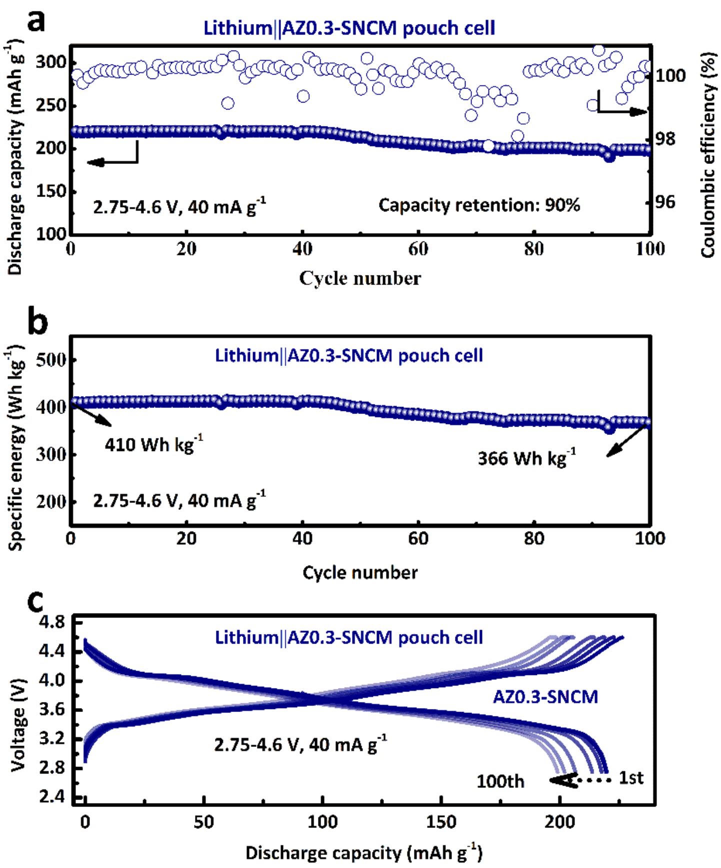

Supplementary Figure 13. Discharge capacity (a), specific energy (b) and charge/discharge curves (c) for 2.04 Ah pouch-type full-cell of undoped SNCM or AZ0.3-SNCM as cathode and Li-foil as anode at different cycles at 0.2 C (40 mA g^{-1}, the grams refer to the cathode active material for all pouch-type full-cell tests, similarly hereinafter) within the voltage range of 2.75-4.6 V at 25°C (the designed specific energy is 410 Wh kg^{-1}, the mass loading of cathode is 40 mg cm^{-2} (ca. 8.4 mAh cm^{-2}) on both sides.).

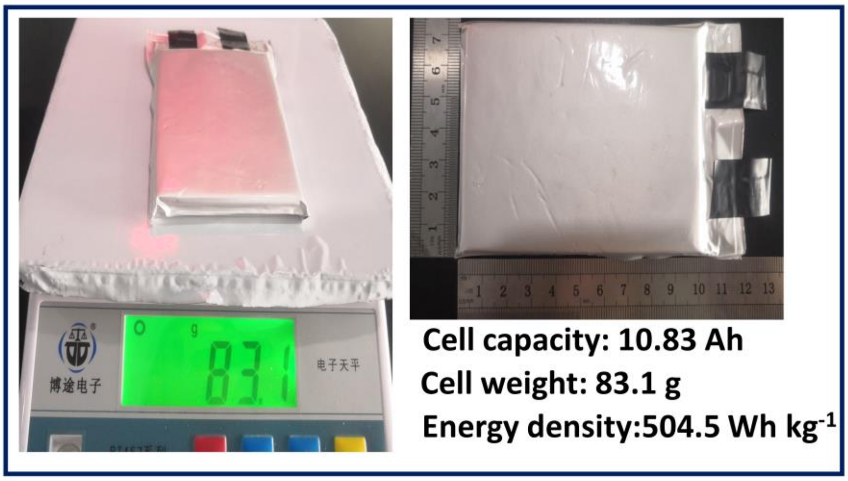

Supplementary Figure 14. Digital image of the 10.83 Ah pouch-type full-cell of AZ0.3-SNCM as cathode and Li-foil as anode in the voltage range of 2.75-4.6 V at 25°C.

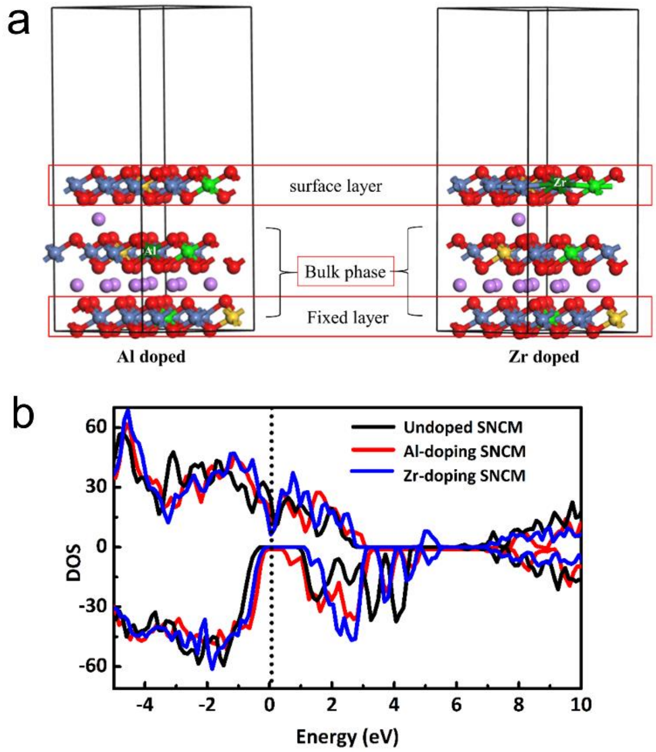

Supplementary Figure 15. The detail model employed in DFT calculation (a). The density of states plots of undoped SNCM, Al doping and Zr doping SNCM (b).

As indicated in Supplementary Figure 15a, it is clearly shown the Al doped in bulk and Zr doped in surface. The top layer is defined as surface, while the other layers far away from vacuum layer is defined as bulk phase. To model the bulk phase, the bottom layer is fixed with the bulk lattice.

The effect of Al and Zr doping on the electronic conductivity and the density of states (DOS) of undoped SNCM and Al or Zr-doped SNCM is shown in Supplementary Figure 15b, it indicates that the conductivity of both Al-SNCM and Zr-SNCM are much higher than that of undoped SNCM.

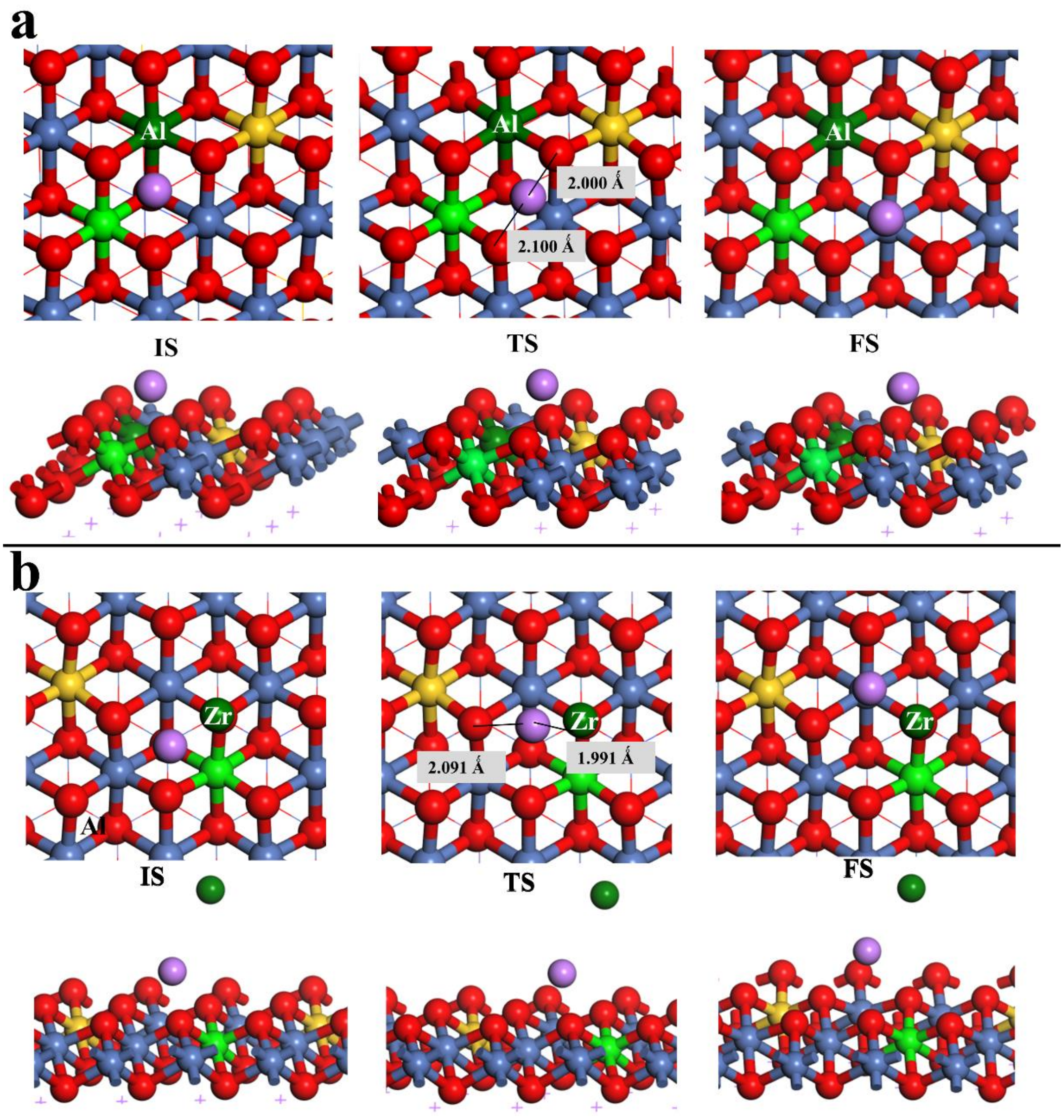

Supplementary Figure 16. Li migration pathway for Al doped SNCM (a) and Zr doped SNCM (b).

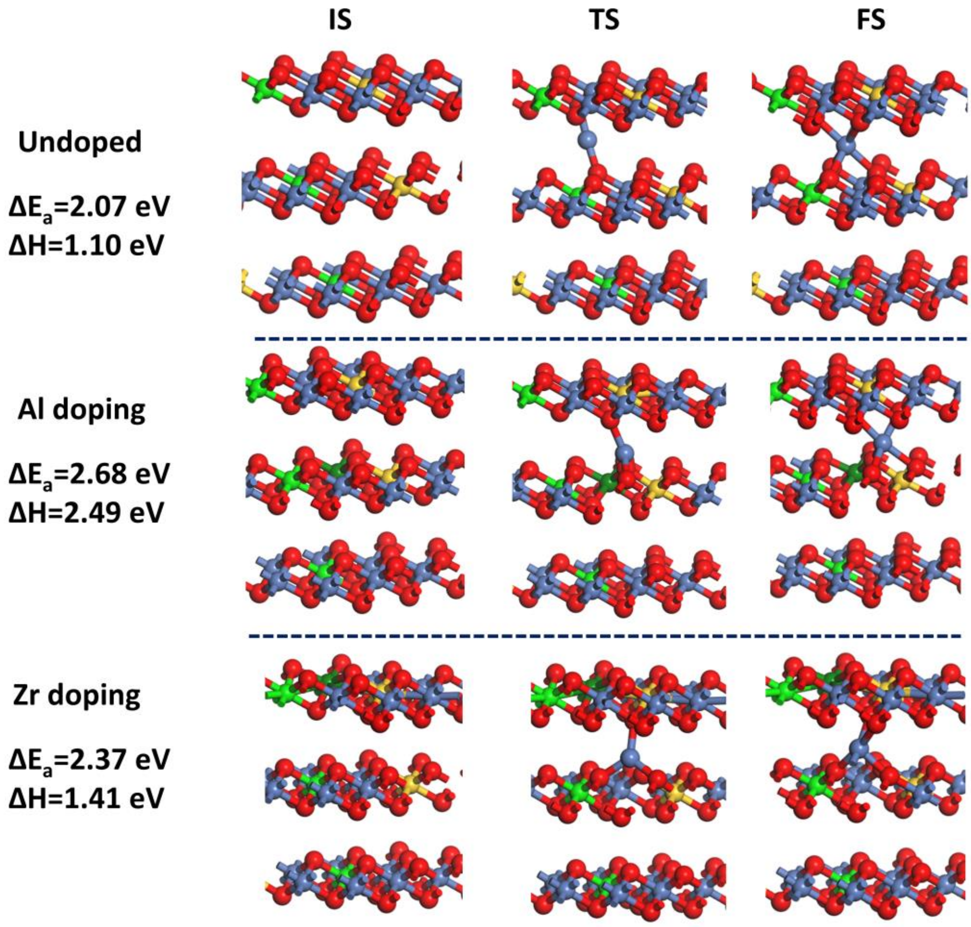

Supplementary Figure 17. The values of migration energy (ΔE_a) and reaction energy difference (ΔH) for undoped SNCM, Al doped SNCM and Zr doped SNCM .

For the migration of Ni, Ni atom in the bulk phase will be out of the internal layer (initial state) and move to gap between the two layers (finial state). It is seen that the barrier for Ni atom migration in undoped SNCM is 2.07 eV, which is lower than those of Zr-doped (2.37 eV) and Al-doped (2.68 eV) configuration. This suggests that the Ni will be more stable after Zr or Al atom being doped.

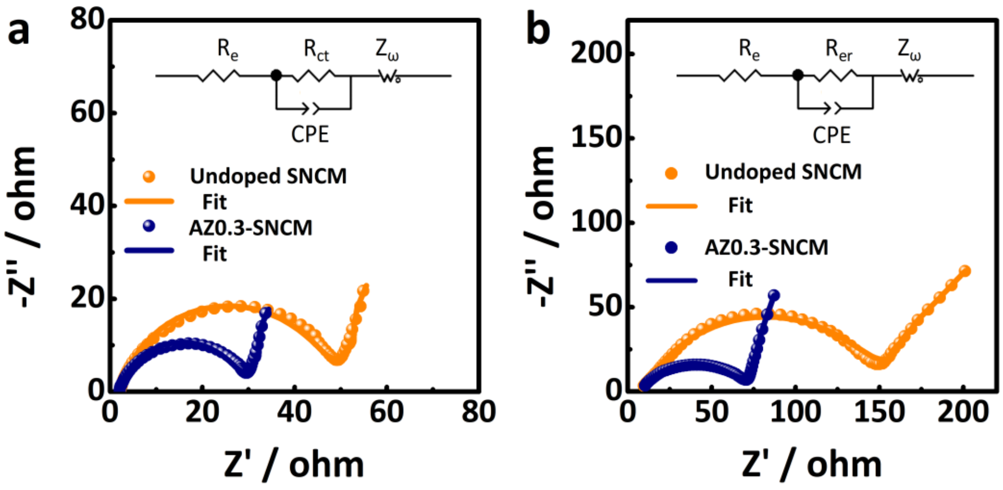

Supplementary Figure 18. Nyquist plots of undoped SNCM and AZ0.3-SNCM cathodes before cycling (a) and after cycling (b).

EIS test was measured at coin-type half-cell at 25 °C, where Li metal anode and 1 M $LiPF_6$ in EC/DEC (1:1 in volume) were used as the counter electrode and electrolyte, respectively. The equivalent circuit modelling is applied to fit the EIS spectra, which includes 4 parts:

(1) The electrolyte resistance (R_e).

(2) The charge transfer resistance (R_{ct}) before cycling (Supplementary Figure 18a) and the combined resistance R_{er} after cycling (Supplementary Figure 18c), which is consisted of R_{ct} (charge transfer resistance) and R_{SEI} (SEI films resistance).

(3) The Warburg impedance representing Li^+ diffusion (Z_ω).

(4) Constant phase angle element (CPE), including the CPE-P and CPE-T.

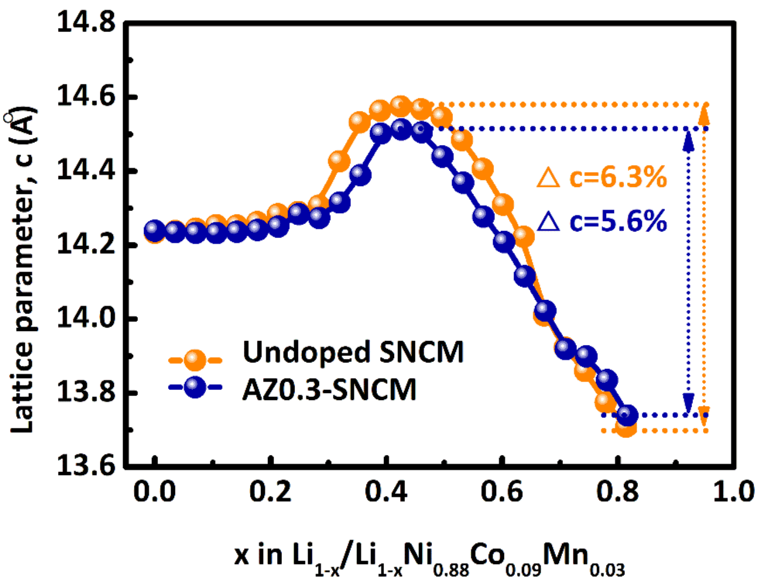

Supplementary Figure 19. The *c*-axis lattice parameters as a function of the cell voltage derived from in situ XRD testing results for undoped SNCM and AZ0.3-SNCM cathodes during the first electrochemical cycling.

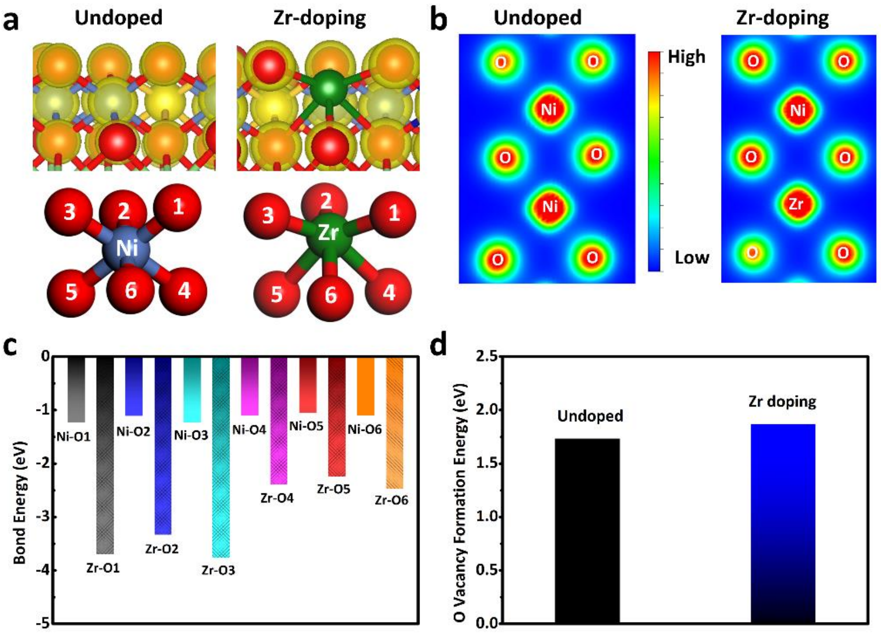

Supplementary Figure 20. Charge distribution scheme and NiO_6 octahedron in undoped SNCM and ZrO_6 octahedron in AZ0.3-SNCM (a). Contour maps of charge density on corresponding planes in undoped SNCM and AZ0.3-SNCM (b). Bond energies of O to Ni and Zr in AZ0.3-SNCM (c). O vacancy formation energy for undoped SNCM and AZ0.3-SNCM, respectively (d).

To demonstrate the charge transference on the Zr-segregated surface of AZ0.3-SNCM, Bader atomic charge analysis has been introduced for the six O atoms in MO_x (M= Zr and Ni) octahedron. As displayed in Supplementary Figure 20a, the charge around O in ZrO_6 octahedron is more negative than that in NiO_6 octahedron, indicating that Zr−O bonds can provide additional negative valence change to O atoms, alleviate the oxidation of O for charge compensation and steady the lattice O^{2-} at the highly delithiation state.[28] This result is confirmed by the contour plots of total charge density in undoped SNCM and AZ0.3-SNCM (Supplementary Figure 20b), revealing that Zr substitution offers extra electrons to O with strong stablization effect. Meanwhile, the much higher bond energy of Zr-O than that of Ni-O in AZ0.3-SNCM and O vacany formation energy in AZ0.3-SNCM than that in undoped SNCM illustrate that Zr can effectively stabilize the structural O with suppression of O_2 release at the high delithiation state (Supplementary Figure 20c). It indicates that the Zr-segregated surface is greatly helpful to enhance its structural stability and H2-H3 transition reversibility during cycling.

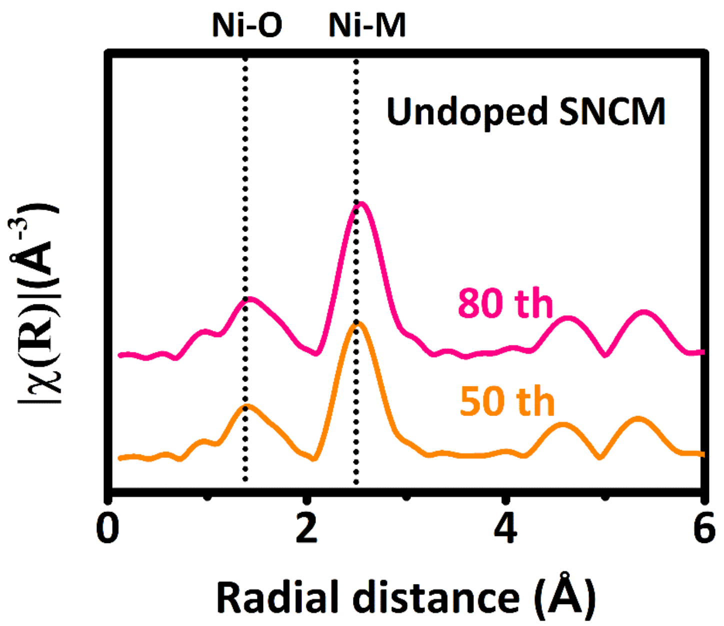

Supplementary Figure 21. Ex situ Ni K-edge EXAFS of undoped SNCM cathode after 80 cycles at 0.5 C (100 mA g^{-1}) and 4.6 V cut-off.

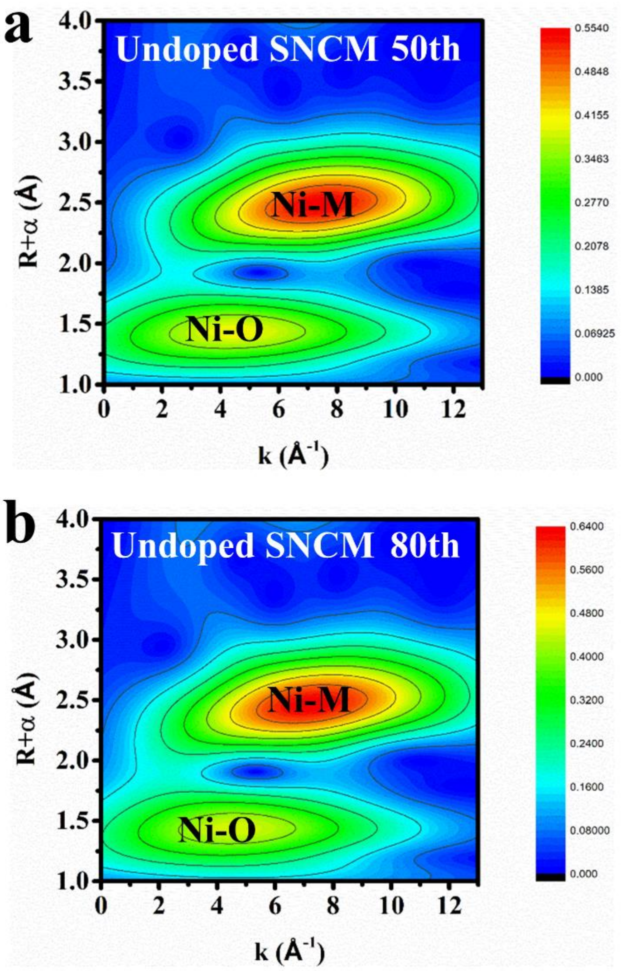

Supplementary Figure 22. The Wavelet Transform EXAFS analysis for undoped SNCM cathode after 50 (a) and 80 (b) at 0.5 C (100 mA g^{-1}) and 4.6 V cut-off.

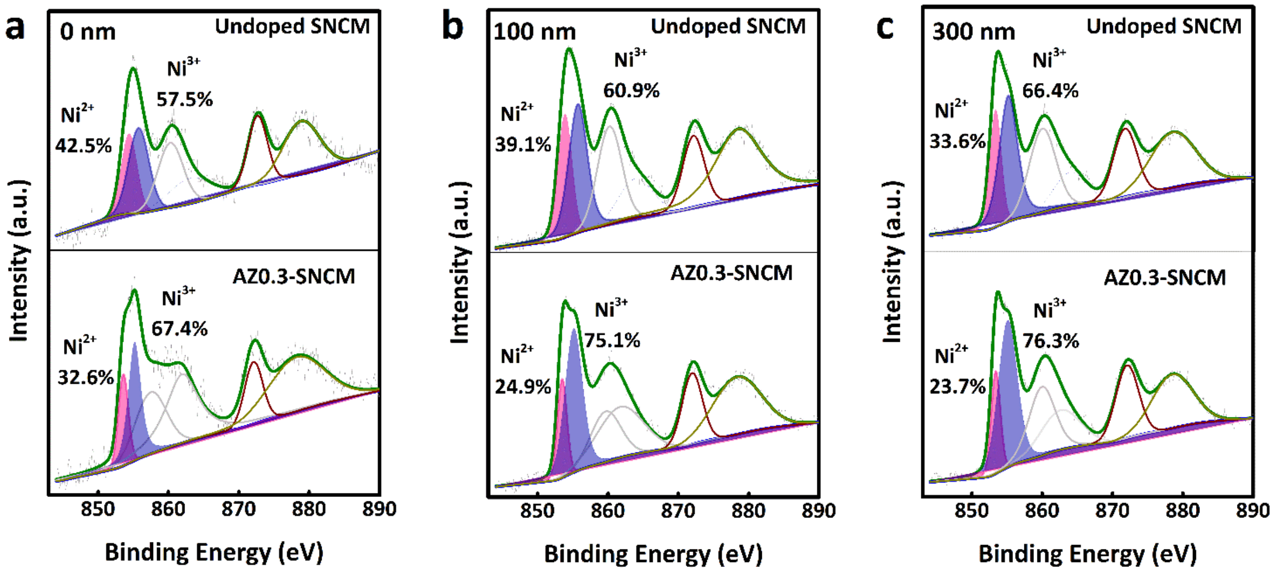

Supplementary Figure 23. Corresponding Ni *2p* XPS spectra on the surface of SNCM and AZ0.3-SNCM after 150 cycles at 0.5 C (100 mA g^{-1}) and 4.6 V cut-off obtained with increasing of Ar^+ etching from XPS depth profiles of 0 nm (a), 100 nm (b) and 300 nm (c).

As indicated in Supplementary Figure 23, it is obvious to identify the heterogeneity distribution of Ni^{3+} from the particle surface towards the center. Specifically, undoped SNCM exhibits the lowest Ni^{3+} concentration at particle periphery (57.5%, Supplementary Figure 23a), it increases gradually to 60.9% in the inner layer (Supplementary Figure 23b), and finally reaches to 66.4% in bulk SNCM (Supplementary Figure 23c). This hierarchical distribution of Ni^{3+} induced by the irreversible H2-H3 phase transition leads to a higher content of Ni^{2+} in SNCM, indicating the instable structure and increased Li/Ni cation mixing within undoped SNCM during cycling. In comparison, AZ0.3-SNCM exhibits a homogenous distribution of Ni^{3+} across the particles. It is noted that the lowest Ni^{3+} concentration in AZ0.3-SNCM (67.4%) is still much higher than the highest Ni^{3+} concentration in undoped SNCM (66.4%). This phenomenon is ascribed to the Jahn-Teller distortion of Ni^{3+}, resulting in the excess of Ni^{2+} and aggravated irreversible H2-H3 phase transition in undoped SNCM. The irreversible phase transition upon cycling within the voltage range of 2.75-4.6 V will lead to the continual structure transition from layered to rock salt phase, which is confirmed by in situ XRD testing results of undoped SNCM (Figure 4c).

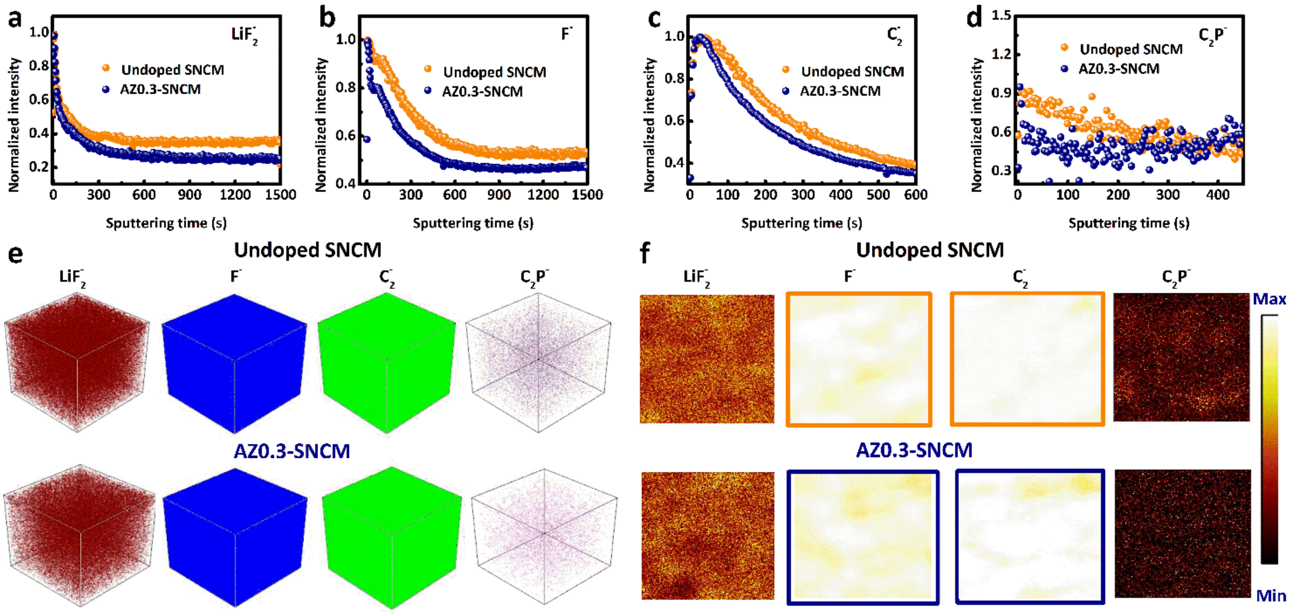

Supplementary Figure 24. TOF-SIMS depth profiles of LiF_2^- (a), F^- (b), C_2^- (c), and C_2P^- (d) species. (e) 3D render of composition (LiF_2^-, F^-, C_2^-, and C_2P^-), concentration distribution (e), and TOF-SIMS chemical imaging of LiF_2^-, F^-, C_2^- and C_2P^- species (f) on undoped SNCM and AZ0.3-SNCM surface after 150 cycles at 0.5 C within 2.75-4.6 V.

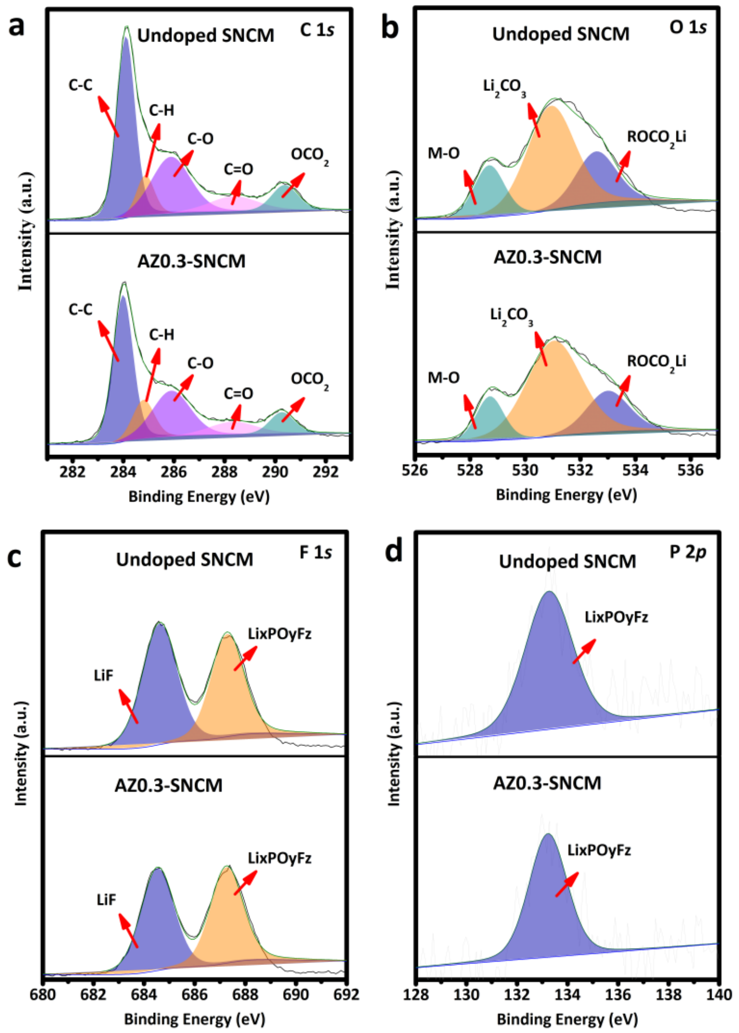

Supplementary Figure 25. The XPS spectra of C *1s* (a), O *1s* (b), F *1s* (c) and P *2p* (d) for cycled undoped SNCM and AZ0.3-SNCM cathodes harvested with a cutoff voltage of 4.6 V after 150 cycles.

The chemical species on cycled cathodes and anodes were further evidenced by X-ray photoelectron spectroscopy (XPS). For F *1s* spectra (Supplementary Figure 25c), the obvious decreased content of $Li_xPO_yF_z$ (686.8 eV) and LiF (684.8 eV) for cycled AZ0.3-SNCM implies the fewer decompositions of electrolyte $LiPF_6$ with generation of thin CEI film, which is in agreement with the TOF-SIMS results (Figure 6). Therefore, delicately-designed dual-doping can alleviate the irreversible phase transition and growth of CEI at high-voltage, thus reducing the formation of spinel structure during the prolong cycling, which exhibits sluggish Li^+ diffusivity.

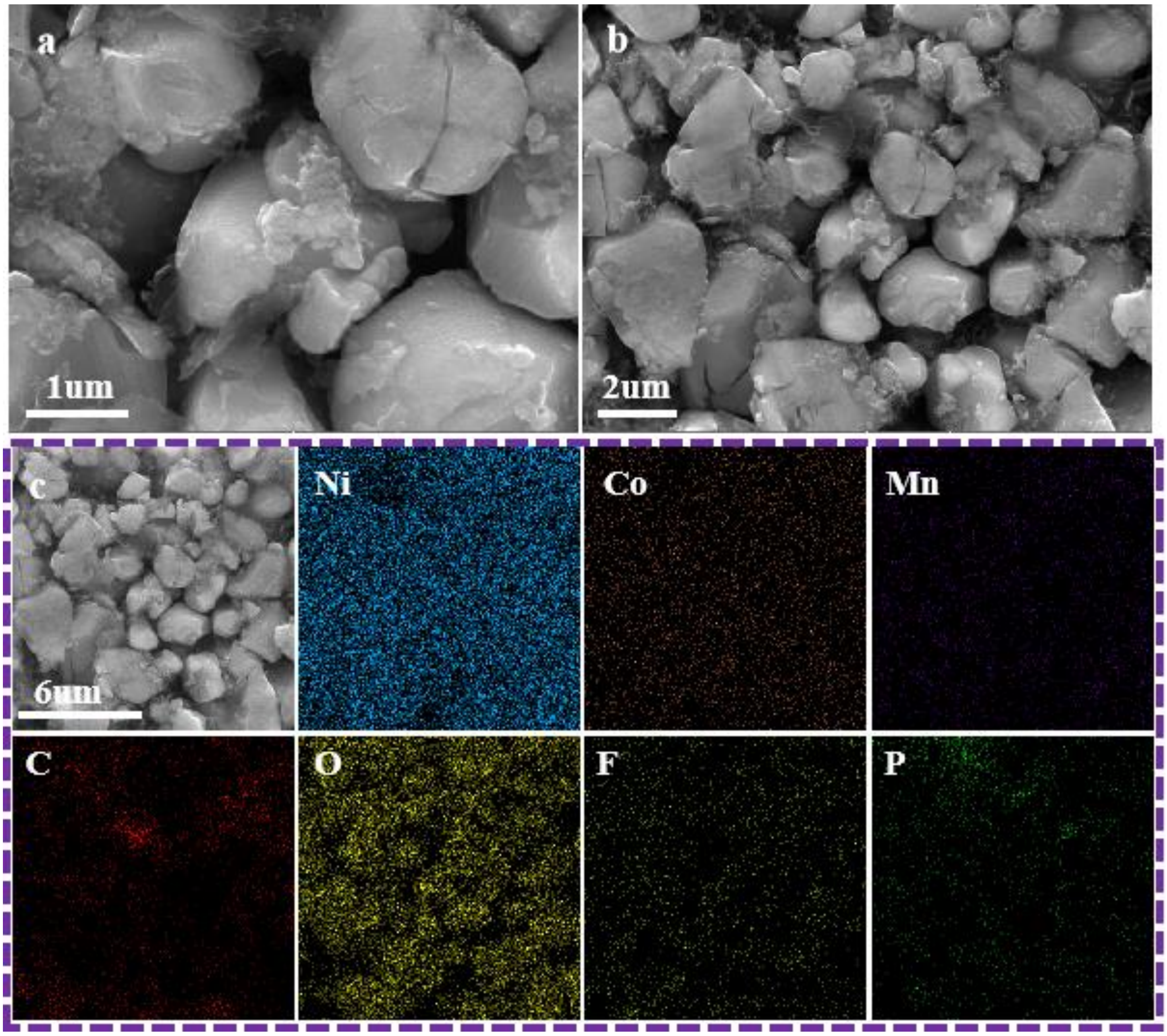

Supplementary Figure 26. SEM images (a, b) and EDS mapping (c) of Ni, Co, Mn, C, O, F and P elements for undoped SNCM electrode after 150 cycles at 0.5 C within 2.75-4.6 V.

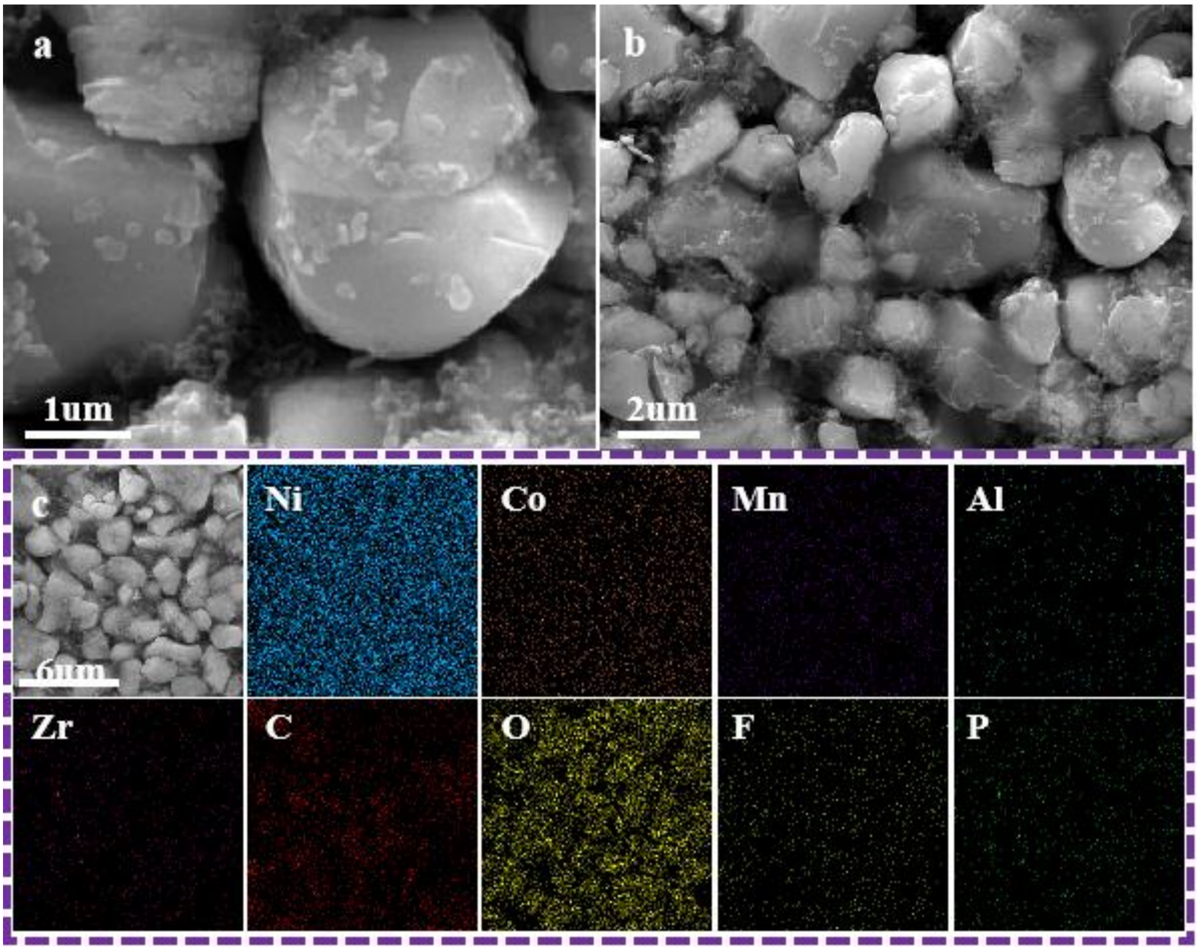

Supplementary Figure 27. SEM images (a, b) and EDS mapping (c) of Ni, Co, Mn, C, O, F and P elements for AZ0.3-SNCM electrode after 150 cycles at 0.5 C within 2.75-4.6 V.

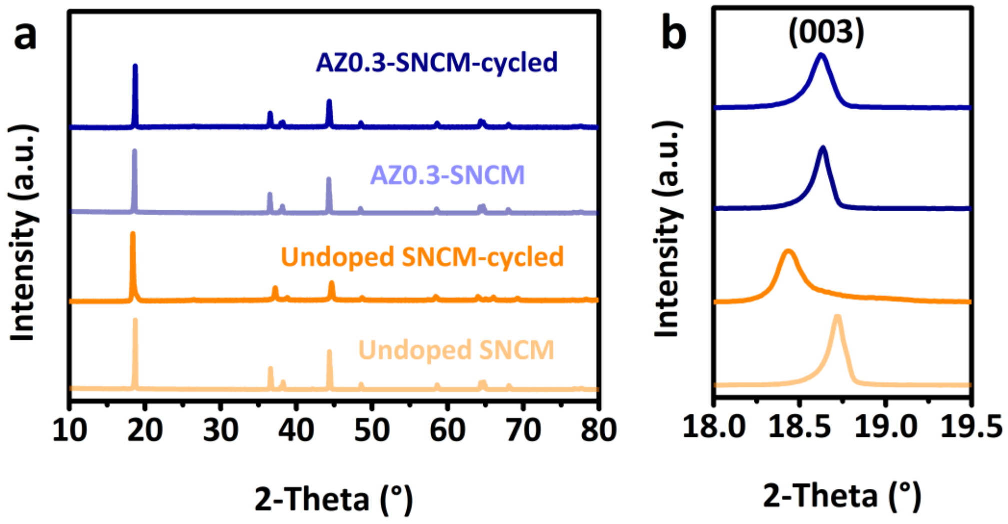

Supplementary Figure 28. Ex situ XRD diffraction recorded for electrodes based on SNCM and AZ0.3-SNCM after 150 cycles at 0.5 C within 2.75-4.6 V (a), and corresponding the enlarged view from 18° to 19.5° (b).

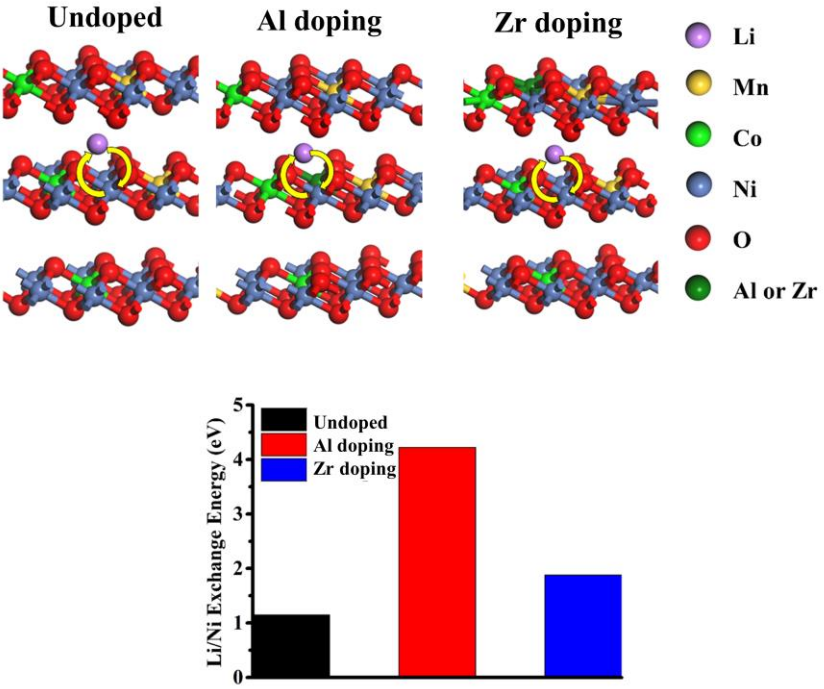

Supplementary Figure 29. Li/Ni exchange energy of undoped SNCM, Al doping and Zr doping SNCM at high delithiated state.

Supplementary Table 1. Chemical compositions of the fabricated cathode materials measured by inductively coupled plasma (ICP) analysis.

Sample	Chemical composition (at. %)				
	Ni	Co	Mn	Al	Zr
Undoped SNCM	87.98	9.08	2.94	-	-
Al-SNCM	87.59	9.22	2.88	0.31	-
Zr-SNCM	87.91	9.06	2.75	-	0.29
AZ0.3-SNCM	87.46	9.09	2.79	0.35	0.31
AZ0.6-SNCM	86.73	8.98	3.04	0.66	0.58
AZ3.0-SNCM	82.46	8.74	2.58	2.90	3.05

Supplementary Table 2. Rietveld refinement results of the undoped SNCM and Al-SNCM samples.

Formula				Undoped SNCM	
Space group				R-3m	
		x	*y*	*z*	*g*
Li1	3b	0	0	1/2	0.980(1)
Ni2	3b	0	0	1/2	0.019(9)
Li2	3a	0	0	0	0.010(0)
Mn	3a	0	0	0	0.030(0)
Co	3a	0	0	0	0.090(0)
Ni1	3a	0	0	0	0.860(0)
O	6c	0	0		1
a-axis/Å				2.875(3)	
c-axis/Å				14.207(6)	
Volume/Å^3				101.725(8)	
R_{wp}/%				2.79	
R_e/%				0.98	
Formula				Al-SNCM	
Space group				R-3m	
		x	*y*	*z*	*g*
Li1	3b	0	0	1/2	0.983(3)
Ni2	3b	0	0	1/2	0.0167(0)
Li2	3a	0	0	0	0.010(0)
Mn	3a	0	0	0	0.030(0)
Co	3a	0	0	0	0.090(0)
Ni1	3a	0	0	0	0.860(0)
Al	3a	0	0	0	0.030(0)
O	6c	0	0		1
a-axis/Å				2.875(2)	
c-axis/Å				14.207(6)	
Volume/Å^3				101.716(4)	
R_{wp}/%				2.69	
R_p/%				0.97	

Supplementary Table 3. Rietveld refinement results of the Zr-SNCM and AZ0.3-SNCM samples.

Formula		Zr-SNCM			
Space group		R-3m			
		x	*y*	*z*	*g*
Li1	3b	0	0	1/2	0.984(0)
Ni2	3b	0	0	1/2	0.016(0)
Li2	3a	0	0	0	0.010(0)
Mn	3a	0	0	0	0.030(0)
Co	3a	0	0	0	0.090(0)
Ni1	3a	0	0	0	0.8610(0)
Zr	3a	0	0	0	0.030(0)
O	6c	0	0		1
a-axis/Å		2.876(9)			
c-axis/Å		14.213(2)			
Volume/$Å^3$		101.879(0)			
R_{wp}/%		2.79			
R_p/%		1.04			
Formula		AZ0.3-SNCM			
Space group		R-3m			
		x	*y*	*z*	*g*
Li1	3b	0	0	1/2	0.984(2)
Ni2	3b	0	0	1/2	0.0158(0)
Li2	3a	0	0	0	0.010(0)
Mn	3a	0	0	0	0.030(0)
Co	3a	0	0	0	0.090(0)
Ni1	3a	0	0	0	0.858(0)
Al	3a	0	0	0	0.030(0)
Zr	3a	0	0	0	0.030(0)
O	6c	0	0		1
a-axis/Å		2.876(1)			
c-axis/Å		14.210(8)			
Volume/$Å^3$		101.802(5)			
R_{wp}/%		2.71			
R_p/%		1.03			

Supplementary Table 4. Comparison of the cycle stability of AZ0.3-SNCM cathode materials with the Ni-rich based materials as reported elsewhere for pouch full cells.

Sample	Voltage range (anode)	Cell capacity	1st capacity (mAh g^{-1})	Current (mA g^{-1})	N/P ratio	Cathode areal capacity (mAh cm^{-2})	Specific energy (Wh kg^{-1})	Cycle Numbers	Capacity retention (%)	Ref.
**AZ-SNCM88**	**2.75-4.6V (Li metal)**	**10.83 Ah**	**225**	**20**	**2.39**	**12.6**	**504.5**	**30**	**86.7**	**Our Work**
**AZ-SNCM88**	**2.75-4.6V (Li metal)**	**2.04 Ah**	**220.8**	**40**	**2.39**	**12.6**	**410**	**100**	**90**	**Our Work**
NCM622	3.0-4.2V (Li metal)	NA	195	270	NA	3.2	482.6	80	95	1
NCM622	2.8-4.4V (Li metal)	CR2032	190	60/200	13.5	3.8	409.9	65	50	2
NCM622	2.7-4.4V (Li metal)	1.2Ah	185	20/60	2.63	3.8	407.9	350	>90	3
NCM811	2.7-4.3V (Li metal)	<1Ah	170	80/800	5	4	387.6	120	>95	4
**AZ-SNCM88**	**2.75-4.55V (graphite)**	**1.8 Ah**	**208.4**	**100**	**1.15**	**6.51**	**250**	**200**	**93**	**Our Work**
**AZ-SNCM88**	**2.75-4.3V (graphite)**	**1.8 Ah**	**198.6**	**40**	**1.15**	**6.51**	**220**	**400**	**92**	**Our Work**
NCM622	2.75-4.4V (Li-C)	NA	180	67	4.04	1.56	325.5	500	82	5
NCM751515	2.5-4.4V (graphite)	NA	190	200/200	1.1	4-4.5	190	600	82	6
NCM811	2.8-4.3V (graphite)	NA	187	100/100	NA	10.6	190	295	80	7
NCA88	2.8-4.2V (graphite)	NA	188	200/200	NA	NA	200	1000	83.40	8
NCMA89	3.0-4.2V (graphite)	0.034Ah	190	200/200	1.15-1.20	NA	200	1000	84.5	9
NCM76	2.5-4.4V (graphite)	NA	179.3	60/60	NA	NA	180	500	89.00	10
SNCM811	2.5-4.4V (graphite)	1.1 Ah	NA	100/100	NA	NA	NA	1000	~80.00	11

Supplementary Table 5. Fitting results for undoped SNCM and AZ0.3-SNCM samples obtained from EIS results.

Samples	$R_s(\Omega)$	$R_{ct}(\Omega)$	$D_{Li}(cm^2\ s^{-1})$	CPE-T	CPE-P
Undoped SNCM-fresh	2.13(5)	41.4(3)	$1.1(7)\times10^{-14}$	$1.3522(3)\times10^{-5}$	0.8361(1)
AZ0.3-SNCM-fresh	2.00(5)	26.3(7)	$5.4(9)\times10^{-14}$	$2.0138(3)\times10^{-5}$	0.8515(7)
Undoped SNCM-after cycling	8.67(4)	143.0(1)	$2.9(7)\times10^{-15}$	$2.7036(9)\times10^{-5}$	0.6931(7)
AZ0.3-SNCM-after cycling	7.24(9)	62.8(3)	$2.3(5)\times10^{-14}$	$5.3231(1)\times10^{-5}$	0.6092(9)

References

1. Han B. *et al.* 500 Wh kg^{-1} class Li metal battery enabled by a self-organized core-shell composite anode. *Adv. Mater.* **32**, 2004793 (2020).

2. Chen S. *et al.* High-Efficiency lithium metal batteries with fire-retardant electrolytes. *Joule* **2**, 1548-1558 (2018).

3. Niu C.J. *et al.* High-energy lithium metal pouch cells with limited anode swelling and long stable cycles. *Nat. Energy* **4**, 551-559 (2019).

4. Kim M.S., *et al.* Langmuir–Blodgett artificial solid-electrolyte interphases for practical lithium metal batteries. *Nat. Energy* **3**, 889-898 (2018).

5. Niu C. *et al.* Self-smoothing anode for achieving high-energy lithium metal batteries under realistic conditions. *Nat. Nanotechnol.* **14**, 594-601 (2019).

6. Oh P., Oh S.-M., Li W., Myeong S., Cho J., Manthiram A. High-Performance heterostructured cathodes for lithium-ion batteries with a Ni-rich layered oxide core and a Li-rich layered oxide shell. *Adv. Sci.* **3**, 1600184 (2016).

7. Beltrop K. *et al.* Triphenylphosphine oxide as highly effective electrolyte additive for graphite/NMC811 lithium ion cells. *Chem. Mater.* **30**, 2726-2741 (2018).

8. Ryu H-H. *et al.* A highly stabilized Ni-rich NCA cathode for high-energy lithium-ion batteries. *Mater. Today* **36**, 73-82 (2020).

9. Kim U.-H., Kuo L.-Y., Kaghazchi P., Yoon C.S., Sun Y.-K. Quaternary layered Ni-rich NCMA cathode for lithium-ion batteries. *ACS Energy Lett.* **4**, 576-582 (2019).

10. Liao J.Y., Oh S.M., Manthiram A. Core/Double-Shell type gradient Ni-rich $LiNi_{0.76}Co_{0.10}Mn_{0.14}O_2$ with high capacity and long cycle life for lithium-ion batteries. *ACS Appl. Mater. Interfaces* **8**, 24543-24549 (2016).

11. Cha H. et al. Boosting reaction homogeneity in high-energy lithium-ion battery cathode materials. *Adv. Mater.* **32**, 2003040 (2020).
